# Effect of Solution pH on the Dual Role of Dissolved Organic Matter in Sensitized Pollutant Photooxidation

**DOI:** 10.1021/acs.est.1c03301

**Published:** 2021-10-29

**Authors:** Jannis Wenk, Cornelia Graf, Michael Aeschbacher, Michael Sander, Silvio Canonica

**Affiliations:** † Eawag, Swiss Federal Institute of Aquatic Science and Technology, CH-8600 Dübendorf, Switzerland; ‡ Institute of Biogeochemistry and Pollutant Dynamics, ETH Zürich, CH-8092 Zürich, Switzerland; § Department of Chemical Engineering and Water Innovation & Research Centre (WIRC), University of Bath, Claverton Down, Bath BA2 7AY, United Kingdom; ∥ INFRAS Research and Consulting, CH-3012 Berne, Switzerland

**Keywords:** excited triplet states, radicals, speciation, reduction potential, photolysis, humic substances, antibiotics

## Abstract

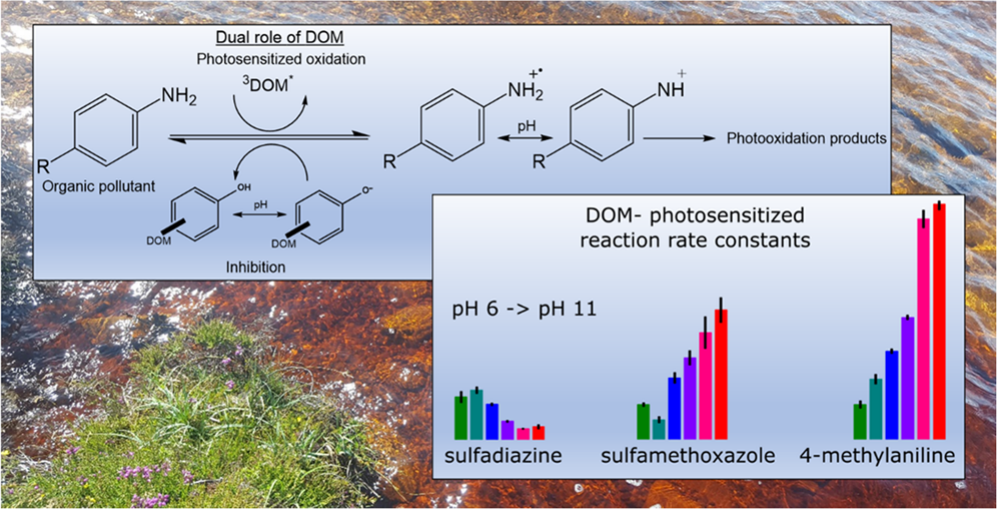

Dissolved organic matter (DOM) has a dual role in indirect phototransformations of aquatic contaminants by acting both as a photosensitizer and an inhibitor. Herein, the pH dependence of the inhibitory effect of DOM and the underlying mechanisms were studied in more than 400 kinetic irradiation experiments over the pH range of 6–11. Experiments employed various combinations of one of three DOM isolates, one of two model photosensitizers, the model antioxidant phenol, and one of nine target compounds (TCs), comprising several aromatic amines, in particular anilines and sulfonamides, and 4-cyanophenol. Using model photosensitizers without antioxidants, the phototransformation of most TCs increased with increasing pH, even for TCs for which pH did not affect speciation. This trend was attributed to pH-dependent formation yields of TC-derived radicals and their re-formation to the parent TC. Analogous trends were observed with DOM as a photosensitizer. Comparison of model and DOM photosensitizer data sets showed increasing inhibitory effects of DOM on TC phototransformation kinetics with increasing pH. In systems with anilines as a TC and phenol as a model antioxidant, pH trends of the inhibitory effect could be rationalized based on the reduction potential difference (Δ*E*_red_) of phenoxyl/phenol and anilinyl/aniline couples. Our results indicate that the light-induced transformation of aromatic amines in the aquatic environment is governed by the pH-dependent inhibitory effects of antioxidant phenolic moieties of DOM and pH-dependent processes related to the formation of amine oxidation intermediates.

## Abstract 2

This study improves the understanding and assessment of organic contaminant abatement that is induced by sunlight in surface waters.

## Introduction

Direct and indirect photochemical reactions are important transformation pathways of biomolecules and contaminants in the aquatic environment,^1−3^ leading, for example, to detoxification of halogenated disinfection byproducts.^4,5^ Dissolved organic matter (DOM) is a chemically complex and structurally diverse component of natural water bodies that plays critical roles in a wide range of environmentally relevant processes.^6−8^ In clear surface waters, DOM is the main absorber of sunlight in the upper water layer. Photon absorption by DOM can trigger both indirect phototransformation of contaminants and photoinactivation of pathogens but also result in DOM photobleaching.^9−18^ Photochemical processes in surface waters involve various short-lived reactive species and different reaction pathways,^19−23^ with DOM and its excited triplet states (^3^DOM*) as key participants.^24−29^ In fact, DOM plays a dual role in photochemical transformation reactions by acting both as a photosensitizer, enhancing photochemical transformations, and as an antioxidant, slowing down photochemical transformations by quenching reactive intermediates.

Excited triplet states, including ^3^DOM*, are generally known to undergo electron-transfer reactions.^30^ According to eq 1, ^3^DOM* may withdraw an electron from an oxidizable organic target compound (TC) to form a DOM radical anion DOM^•–^ and a one-electron oxidized contaminant radical cation TC^•+^. The latter are short-lived intermediates that may further react to stable oxidation products TC_ox_ (eq 2). However, TC^•+^ may itself abstract an electron from an electron-rich antioxidant (AO) moiety, a reaction that reconstitutes the parent TC (eq 3). Reaction with the antioxidant thus quenches TC oxidation.
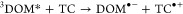
1
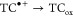
2
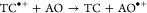
3The inhibition of triplet-induced reactions through antioxidants lowers the rate of TC transformation. Phenolic moieties, which are abundant in DOM,^31,32^ are considered a major reservoir of antioxidant capacity in natural waters.

The effect of DOM and model antioxidants on triplet-induced oxidations of organic compounds has been investigated in several recent studies.^33−42^ These studies showed that DOM inhibits the oxidative transformation of a wide range of environmentally relevant compounds, particularly when containing aniline functional groups, such as sulfonamide antibiotics, and phenolic groups.^33^ The inhibitory effect of terrestrial DOM was found to be substantially higher than that of aquatic DOM.^34^ The antioxidant properties of DOM were characterized using electrochemical techniques, which provided evidence for phenolic moieties within DOM as main antioxidants.^43,44^ The role of phenolic moieties as antioxidants in DOM was further characterized by kinetic irradiation experiments in model systems with triplet photosensitizers and either phenolic antioxidants or DOM as an antioxidant.^35^ Partial preoxidation of DOM by ozone diminished its antioxidant activity^38^ and also decreased the inhibitory effect of DOM on triplet-induced transformations.^36^ Quenching of triplet states by DOM was ruled out as a potential cause of the inhibitory effect.^45^ The inhibitory effect of DOM has been reported for radical cations induced by direct absorption of light.^39^ However, reformation of initial compounds from metastable transformation products may also occur for other types of photochemical reaction pathways, such as reversible photohydrations and photooxygenations.^46−49^

The aims of this study were (1) to investigate how the inhibitory effect of DOM changes with pH and (2) to compare this effect to other pH-dependent effects on triplet-induced transformation kinetics. We anticipated that varying the pH would largely alter the inhibitory effect due to causing speciation shifts of the phenolic antioxidant moieties within the DOM. These phenolic moieties deprotonate to phenolates over a wide pH range centered at ≈9.7.^32,50^ Phenolates are more readily oxidized than undissociated phenols because their one-electron oxidation potential is ≈0.7 V higher compared to their protonated counterparts.^51^ Therefore, the working hypothesis for this study was that the inhibitory effect of DOM, as well as of phenolic model antioxidants, increases with pH from neutral to basic solution pH conditions.

However, this working hypothesis may be simplistic because more complex pH dependencies are conceivable. pH effects on radical intermediates might weaken or even reverse the trend expected based on our working hypothesis. pH affects not only the equilibrium speciation of aniline radical cations,^52,53^ which are key intermediates for the inhibitory effect (see eq 3), but also the rates of the corresponding deprotonation and protonation reactions.^54^ With increasing pH, the deprotonation rates of the radical cation TC^•+^ are expected to increase and the protonation equilibrium is shifted to the deprotonated species. The latter are weaker oxidants than the radical cations, and a decrease in the reduction rate of the radicals and thus a smaller inhibitory effect is expected. Furthermore, TC^•+^ and their deprotonated counterparts are possibly involved in other pH-dependent reactions leading to their further transformation to oxidized products or to reduction back to their parent compounds. Finally, pH may also alter transformation kinetics by changing light absorption characteristics of DOM, production rates and reactivities of ^3^DOM* (generated from a variety of different precursor chromophores), and changes in TC ground-state speciation. Despite the importance of pH on DOM photochemistry in aquatic photochemical reactions, most studies considering the effect of pH on surface water photochemistry focus on indirect photodegradation of specific target compounds while providing an explanatory framework around pH-induced shifts of reactive species steady-state concentration and target compound speciation^55−57^ but without considering photochemical or photophysical processes, such as changes in fluorescence intensity with pH,^58^ within DOM in detail. pH is an important driver in DOM photobleaching, which increases toward both lower and higher pH exhibiting a minimum around pH 6–6.5.^59^ Increased photobleaching at higher pH has been explained with the expansion of DOM and its chromophores,^60^ along with increasing light absorption with increasing pH^61^ and enhanced internal charge transfer due to deprotonated phenolic moieties.^62^

The irradiation experiments for this study were carried out with photosensitizers, antioxidants, and TCs chosen based on previous studies^34−36,51,63,64^ in solutions covering a range of pH 6–11. This pH range was selected to include the pH of most natural surface waters (∼7–9) and to cover protonation equilibria of phenolic moieties of DOM. Lower pH within the range of pH ∼2–6, which are, for example, occurring in atmospheric water^65^ and acid mine drainage,^66^ were not included due to the additional complexity arising by protonation equilibria of TCs and carboxylic moieties within DOM (p*K*_a_ ≈ 4).^32,50^ In the studied pH range, carboxylic moieties of DOM are not expected to play an important role for the investigated kinetic changes because they are mostly deprotonated.

Aromatic ketones and DOM isolates were employed as photosensitizers. Suwannee River fulvic acid (SRFA) and Nordic aquatic fulvic acid (NAFA) served as representative DOMs or terrestrial origin, derived from higher plants, while Pony Lake fulvic acid (PLFA) served as representative aquatic DOM derived mostly from microbial sources.^67^ Anilines, including sulfonamides, and 4-cyanophenol served as TCs. Choice of TCs was based on previous studies characterizing the inhibitory effect of DOM on anilines and sulfonamides at fixed pH,^33−35^ the importance of anilines and their derivatives as aquatic contaminants,^68,69^ including their frequent use as model pollutants,^70^ and the availability of radical cation standard one-electron reduction potential data. Sulfonamides are a broad class of high-usage classic antibiotics with various ecotoxicological effects in the environment that can be frequently detected in surface waters, including at above micromolar concentrations.^71^ 4-Cyanophenol was chosen as a representative phenolic compound without antioxidant properties. Both unsubstituted phenol and DOM isolates were used as antioxidants. Four different types of experimental systems were utilized to study TC phototransformation, namely: System 1, employing either a model photosensitizer or DOM (in the latter case, DOM acted both as a photosensitizer and an antioxidant); System 2, employing a model photosensitizer and DOM as an antioxidant; System 3, employing DOM as a photosensitizer and a model antioxidant; and System 4, employing a model photosensitizer and a model antioxidant.

## Materials and Methods

### Chemicals

A list of target compounds, photosensitizers, antioxidants, and DOMs used in this study is provided in Table 1. Supplier and purity details, including information on additional chemicals used and preparation of stock solutions, are provided in the Supporting Information (SI), Text S1.

**Table 1:** Target Compounds, Model Photosensitizers, Model Antioxidant, and DOM Isolates, with Acid Dissociation Constants (p*K*_a_) and One-Electron Standard Reduction Potentials (*E*_red_^°^) of Their Relevant Reactive Species

	compound	abbreviation	CAS-RN IHSS no.	p*K*_a_^a^	p*K*_a_^* ^b	*E*_red_^°^ c (V vs SHE)
model photosensitizers	2-acetonaphthone	2AN	93-08-3		1.7^72^	1.10,^45,73^ 1.34^74^
	4-carboxbenzophenone	CBBP	611-95-0	4.57^75^	n.a.^76^ d	1.83^45,77^
target compounds	aniline	ANI	62-53-3	4.87^75^	7.05^52^	1.02^53^
	4-methoxyaniline	4MtA	104-94-9	5.36^75^	9.6^53^	0.79^53^
	4-methylaniline	4MA	106-49-0	5.08^75^	8.5^53^	0.92^53^
	*N*,*N*-dimethylaniline	DMA	121-96-7	5.07^75^	n.a.	0.87^78^
	sulfamethoxazole	SMX	723-46-6	1.6 ± 0.2, 5.7 ± 0.2^81^	n.a.	n.a.
	sulfachloropyridazine	SCPD	80-32-0	2 ± 3, 5.9 ± 0.3^81^	n.a.	n.a.
	sulfadiazine	SD	38-35-9	2 ± 1, 6.4 ± 0.6^28^	2.9^82^	1.30^c^, 1.09^82^ e
	4-cyanophenol	4CNP	767-00-0	7.97^75^	<0^83^	1.71^51^ c, 1.12^84^ f
model antioxidant	phenol		108-95-2	9.99^75^	–2^83,85^	1.5^51^ c, 0.79^84^ f
DOM isolates	Suwannee River fulvic acid	SRFA	2S101F	3.76, 9.84^50^	n.a.	n.a.
	Pony Lake fulvic acid	PLFA	1R109F	4.52, 9.48^86^	n.a.	n.a.
	Nordic aquatic fulvic acid	NAFA	1R105F	3.79, 9.67^32^	n.a.	n.a.

a Titration fitting parameters for proton binding of IHSS extracts assuming two main types of proton binding sites within humic substances, namely, carboxylic acids and phenols.^32^

b Dissociation constant of relevant reactive species: protonated excited triplet state (photosensitizer), radical cation (target compound), or protonated phenoxy radical (target compound or model antioxidant).

c Standard one-electron reduction potential of the excited triplet state (photosensitizer) or radical cation (TC^•+^/TC), except where noted (unit: V vs standard hydrogen electrode, SHE).

d n.a.: not available.

e Standard one-electron reduction potential of sulfadiazine radical/sulfadiazine anion (SD^•^/SD^–^).

f Standard one-electron reduction potential of phenoxyl radical/phenolate (PhO^•^/PhO^–^).

### Preparation of Solutions

All aqueous solutions were prepared using ultrapure water (Milli-Q, Millipore). Solutions for irradiations were prepared in 20 mL (final volume) capped quartz-glass tubes with a headspace of approximately 2 mL and contained 5 mM phosphate buffer, which was used throughout the investigated pH range of 6–11. Phosphate-buffered solutions for all experiments were used to avoid possible effects on TC transformation kinetics due to the increasing importance of carbonate radicals in carbonate-buffered systems at elevated pH.^26,55,56^ Note that experiments conducted at pH 9 and 10 were not in the optimum buffer range of phosphate. However, pH measurements before and after each set of irradiations showed that the pH values drifted by ≤0.05 pH units, even under these alkaline conditions. Solutions contained different combinations of a single TC, a model photosensitizer or DOM as a photosensitizer, and a model antioxidant or DOM as an antioxidant. The initial TC concentration was 5 μM. Concentrations of model photosensitizers were 50 μM for CBBP and 10–100 μM for 2AN (Table S5a,b for different TCs). TC, model photosensitizer, and phenol addition to the solutions did not change its pH. The DOM concentration was 2.5 mg C L^–1^ (applied in most cases) or 1.0 mg C L^–1^. Addition of DOM stock solutions decreased the solution pH by 0.1 ± 0.02 units at pH 9–11. Possible changes in TC phototransformation kinetics resulting from this slight pH decrease were assumed to be within the experimental error. Thus, we report target integer pH values in the Results and Discussion section. The concentrations of the model antioxidant phenol were 10 or 25 μM. The added phenol concentration is reasonably comparable to the concentration of both phenolic moieties and electron-donating groups within solutions containing 1–2.5 mg C L^–1^ DOM.^35^ SRFA and NAFA phenolic content has been measured via titration and 1 mg C L^–1^ corresponds to 3 μM phenolic moieties, while not all phenolic moieties act as antioxidants.^32^ Similarly, the electron-donating capacity (EDC) for a variety of humic substances was measured at an applied potential of *E*_h_ = 0.61 V, pH 7, and ranged approximately from 0.6 to 1.8 μM EDC per mg humic substance.^43^

### Irradiation Experiments

The irradiation setup consisted of a merry-go-round photoreactor (Hans Mangels, Germany) that was equipped with a 500 W medium-pressure lamp, a borosilicate glass cooling jacket, a 0.15 M sodium nitrate filter solution which cut off lamp emission wavelengths ≤320 nm, to minimize direct phototransformation of TCs, and a cooling system adjusted to 25 °C (±0.2 °C). Further details on the setup are provided elsewhere.^34^ Model and DOM photosensitizer concentrations and irradiation times for complete kinetics experiments (SI Tables S2, S3, and S5–S8) were optimized according to preliminary trials and results of previous studies,^34−36^ irradiation times ranged from 5 to 90 min. Differences in model photosensitizer concentration across different experiments and pH are not expected to affect normalized reaction rate constants. To determine transformation kinetics of the TCs, six samples of 400 μL were withdrawn from each quartz tube in equidistant time intervals during photoirradiation. Samples were immediately stored at 4 °C and analyzed by high-performance liquid chromatography (HPLC) within 36 h. Most experiments were performed in duplicate (a few in triplicate) and were found to yield reproducible results with a deviation in reaction rate constants of <10% between repetition experiments, except for a few experiments conducted at pH 10 and 11 with higher deviations. Details on analytical methods, including HPLC analysis, UV–vis absorption, and pH-measurements are provided in Text S2 and Table S1.

### Data Analysis

The depletion of TCs was fitted assuming pseudo-first-order reaction kinetics (i.e., reaction rate constants *k* (s^–1^) equalled the linear slopes of TC concentrations, expressed in natural logarithmic units, vs irradiation times). To achieve comparable results across all TC/photosensitizer/inhibitor/pH combinations, reaction rate constants were first corrected for direct phototransformation of the TC (see Text S3 for discussion on the effect of pH on direct phototransformation for single TCs and Tables S2 and S3 for measured direct phototransformation rate constants) and for System 2 experiments (i.e., for solutions with model photosensitizers and DOM as an antioxidant), to compensate for the photosensitizing effect of DOM. These corrections were performed separately for separate pH values by subtracting reaction rate constants for each TC in pure water or in solutions containing only DOM without model sensitizers from rate constants obtained with model sensitizers (either in the absence or presence of DOM). Light-screening correction was applied in a second step using the factors for different photosensitizer and inhibitor combinations listed in Tables S9–S14. Details on the determination of correction factors are provided elsewhere.^36^ Consistent with previous publications, the corrected rate constants are labelled with the superscript (2) (i.e., *k*^(2)^) to indicate that two corrections were made.^34−36^ In no case did the corrections alter trends that were apparent already in the uncorrected data. UV-absorption spectra of TCs and phenol are shown in Figures S1 and S2. UV–vis absorption spectra of model and DOM photosensitizers, molar (model photosensitizers), and specific absorption coefficients (DOM) at the relevant wavelengths of the irradiation system are provided in Figure S4 and Table S4, alongside a discussion on the effect of spectral changes of both model photosensitizers and DOM on the observed rate constants in this study, as well as the importance of pH-induced changes in photophysical processes (Text S4).

## Results and Discussion

### Transformation of TCs by Model Photosensitizers and DOM (System 1)

**Figure 1:**
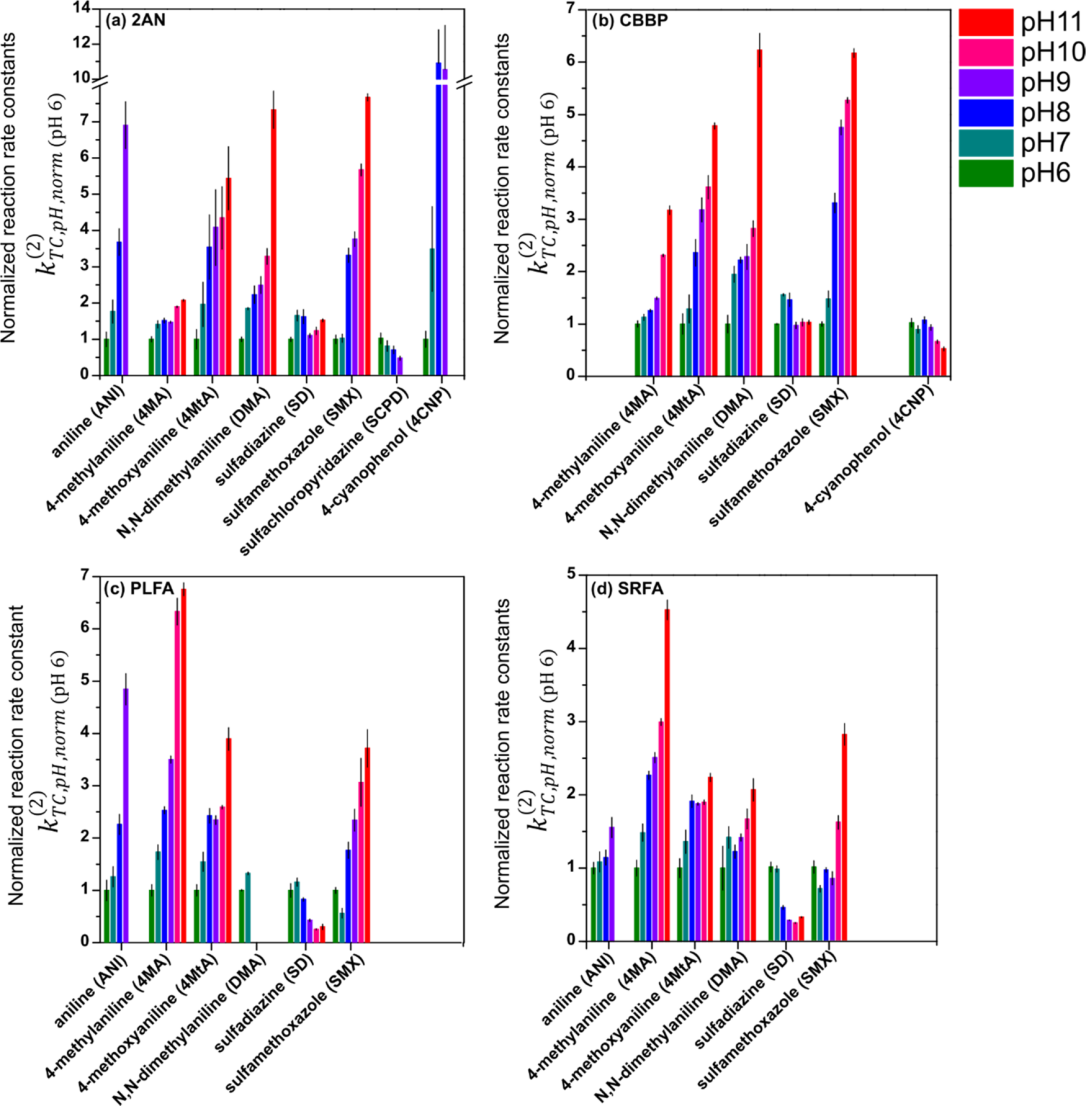
Pseudo-first-order rate constants, normalized to the corresponding values at pH 6, for the transformation of target compounds photosensitized by model photosensitizers (a) 2-acetonaphthone (2AN), (b) benzophenone-4-carboxylate (CBBP), or dissolved organic matter isolates, (c) Pony Lake fulvic acid (PLFA), and (d) Suwannee River fulvic acid (SRFA) at several different pH values indicated in the legend. Error bars show 95% confidence intervals (Tables S4–S8).

We determined the rate constants of indirect phototransformation for nine TCs in combination with two model photosensitizers, 2AN (Figure 1a) and CBBP (Figure 1b), and two DOM isolates, PLFA (Figure 1c) and SRFA (Figure 1d) over the pH range of 6–11. To facilitate comparison between different TCs and photosensitizers, the displayed rate constants, *k*_TC,pH,norm_^(2)^, were corrected rate constants (*k*_TC,pH_^(2)^) normalized to the value obtained at the lowest tested pH of 6 (*k*_TC,pH 6_^(2)^), i.e., *k*_TC,pH,norm_^(2)^ = *k*_TC,pH_^(2)^/*k*_TC,pH 6_^(2)^. Numerical values of all of these rate constants are provided in Tables S2, S3, and S5–S8, in the SI.

The rate constants in systems containing a model sensitizer (either 2AN or CBBP) reflect only the effect of pH on photosensitized TC transformation as there were no antioxidants in these solutions, and thus no inhibitory effects. Rate constants of TC phototransformation in the presence of PLFA and SRFA reflect the combined effect of DOM photosensitization and inhibitory effects through DOM antioxidant moieties. Concerning the effect of reactive oxygen species with changing pH, Text S5 discusses the role of superoxide and in detail the possible impact of singlet oxygen (^1^O_2_) on the observed rate constants. In summary, the impact of ^1^O_2_ is expected minor based on its available reaction rate constants with TCs.

In the systems containing the model photosensitizers 2AN or CBBP, the transformation rate constants of anilines (ANI, 4MA, 4MtA, and DMA) strongly increased with increasing pH, between a factor of 2.1 (4MA) and 9.3 (DMA), relative to the lowest rate constant measured at pH 6. This pH trend for anilines may be rationalized by aniline radical cations formed through one-electron oxidation deprotonating at higher pH. For ANI, 4MA, and 4MtA, the resulting anilinyl radicals are expected to be more prone to coupling reactions, which would compete with a possible reduction by superoxide (Text S5). For DMA, deprotonation of the radical cation would lead to a carbon-centered radical on one of the methyl groups and further degradation, also competing with a possible reduction by superoxide (Text S5). As compared to the anilines, there was no consistent pH trend for the sulfonamides SMX, SCPD, and SD. With increasing pH, the phototransformation rate constants strongly increased for SMX, moderately decreased for SPCD, while no clear pH trend was observed for SD.

When comparing transformation by the two photosensitizers 2AN and CBBP, the effect of pH on the normalized pseudo-first-order rate constants for each individual TC was similar with a single exception: For 4CNP (p*K*_a_: 7.97), the normalized rate constants strongly increased with pH for 2AN but not for CBBP. This observation can be explained by the different one-electron reduction potentials of the photosensitizers in their excited triplet states (*E*_red_^0 *^(^3^Sens*/Sens^•–^)). Assuming that the phototransformation of 4CNP is initiated by a one-electron transfer to the excited triplet state of the photosensitizer,^51^ excited triplet CBBP (*E*_red_^0 *^(^3^CBBP*/CBBP^•–^) = 1.83 V vs standard hydrogen electrode (SHE)) is expected to undergo a diffusion-controlled reaction with both the undissociated and deprotonated forms of 4CNP (having one-electron oxidation potentials of −1.71 and −1.12 V vs SHE, respectively), which explains the lack of pH effect on the transformation rate of 4CNP. In contrast, for ^3^2AN* with a lower one-electron reduction potential (*E*_red_^0 *^(^3^2AN*/2AN^•–^) = 1.10–1.34 V vs SHE), a fast reaction is only expected to occur with the deprotonated form of 4CNP, explaining the enhancement in the transformation rate of 4CNP with increasing pH.

In qualitative terms, TC reaction rate constants with DOM as a photosensitizer (Figure 1c,d) had a similar pH dependence as those observed with the model photosensitizers. This finding supports that both 2AN and CBBP are suitable model photosensitizers to mimic the ^3^DOM*-induced TC oxidation over the studied pH range. However, compared to the experimental series with model photosensitizers only, we expected decreased photosensitized transformation rate constants with increasing pH due to the presence of DOM antioxidant moieties that inhibit transformation. Given the two competing processes of photosensitization and inhibition in systems containing DOM, the similar pH trends observed in the presence and absence of DOM antioxidant moieties suggest that inhibition by DOM generally played a minor role. The only exception to this conclusion is the reaction of SD: its rate constants were not strongly affected by pH for model sensitizers but decreased with increasing pH when DOM was the photosensitizer. The latter finding concurs with the expectation that the inhibition caused by antioxidant moieties of DOM increases with increasing pH. The absence of a significant pH effect for SD transformation without antioxidants agrees with previous observations.^28^ This difference to the behavior of the anilines and SMX is possibly related to the particular pathway of triplet-sensitized phototransformation of sulfonamides exhibiting a six-membered heterocyclic substituent (such as SD, but not SMX), which leads to the formation of SO_2_ extrusion products.^28^ The limited amount of data available for SCPD appear to indicate a pH dependence of the rate constants more similar to SD than SMX, which concurs with the same type of substituent carried by SD and SCPD.

Note, for DOM as a photosensitizer, electrostatic attraction between negatively charged DOM and TC compounds can be neglected over the whole pH range, since none of the TCs is present as a positively charged (cationic) species at pH ≥6. This includes SD, exhibiting a significant speciation change between pH 6 and 7 from neutral (zwitterionic) to anionic, while changes on the photosensitized rate constant of SD are small in this pH span compared to those observed for pH 7–11.

### Assessing the Individual Inhibition and Photosensitization Contribution in DOM-Induced Phototransformations

Due to the intrinsic coexistence of photosensitizing and antioxidant moieties in DOM, the individual contribution of the two types of moieties can be determined using comparative irradiation experiments. In previous studies, we expressed inhibition by DOM based on a calculated inhibition factor (IF).^34−36^ IF was defined as the ratio of rate constants for the transformation of a TC in the presence and absence of antioxidants obtained with the same photosensitizer (under identical irradiation conditions). Here, we use an analogous concept: the normalized reaction rate constants for the transformation of a TC photosensitized by a given DOM at a certain pH are divided by the corresponding normalized rate constants obtained using a given model photosensitizer (in the absence of inhibitors). This ratio is termed here as “comparative inhibition factor” (CIF), expressed as: CIF = *k*_TC,pH,norm,DOM_^(2)^/*k*_TC,pH,norm,Sens_^(2)^. This parameter is a less precise indicator of the inhibitory effect than IF because it is based on rate constants obtained using two different photosensitizers (i.e., a given DOM and a given model sensitizer). In addition, DOM photosensitizer moieties are subject to changes in absorbance and possibly in intersystem crossing quantum yields with varying solution pH (Texts S4 and S5), while we do not expect such pH effects to occur for the used model photosensitizers.

**Figure 2:**
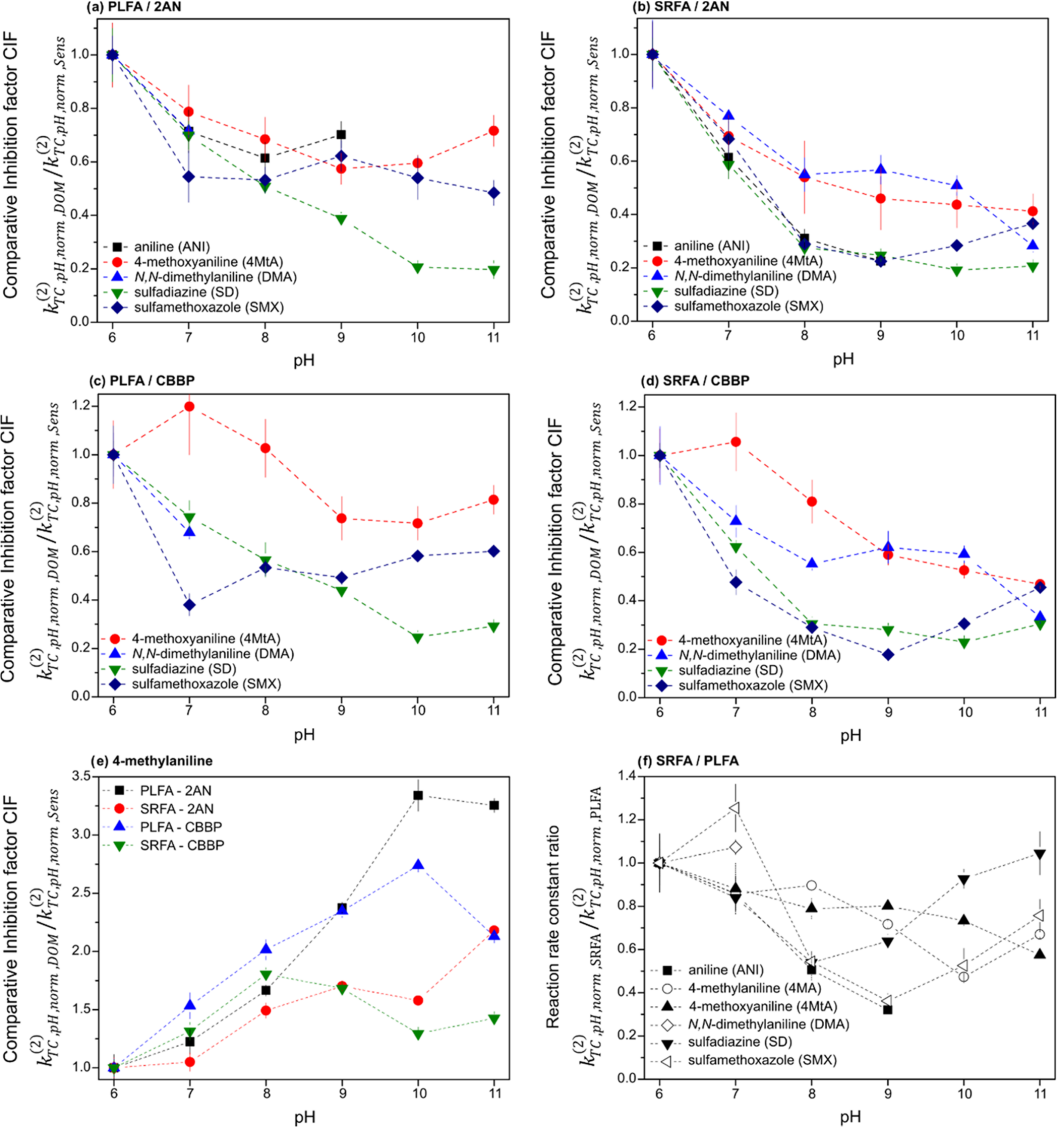
Comparative inhibition factors of photosensitized transformation of target compounds (TCs) for the DOM isolates (2.5 mg C L^–1^) Pony lake fulvic acid (PLFA) and Suwannee River fulvic acid (SRFA) in relation to the model sensitizers 2-acetonaphthone (2AN) and benzophenone-4-carboxylate (CBBP) (a–e) and a reaction rate constant ratio of SRFA and PLFA photosensitized transformations of TCs (f) at different pH. Experimental error bars were determined by the error propagation law using 95% confidence intervals of pseudo-first-order transformation rate constants of single photosensitizer/TC systems.

Figure 2a–e shows calculated CIF of the four DOM-model sensitizer combinations and six TCs. Note that the values of CIF at pH 6 are always 1 (by definition). Therefore, CIF is not able to reveal an inhibitory effect at pH 6 but is useful to describe the changes in inhibitory effect with increasing pH. We used the lowest tested pH value as the reference point for data evaluation primarily based on our initial hypothesis that phenolic antioxidant activity increases with increasing pH. For most target compounds, CIF decreased as the pH increased (Figure 2a–d), supporting the hypothesis that the inhibitory effect of DOM increases with increasing pH because of an increased fraction of deprotonated phenolic moieties in the DOM. We observed the opposite trend for 4MA for which we currently have no explanation (Figure 2e): CIF increased with increasing pH.

When evaluating data sets across different TCs, the decrease in CIF was largest over the circumneutral pH range between pH 6 and 8. At mildly alkaline conditions from pH 8, the increase in inhibition subsided or reversed. Also considering the observed exception for 4MA, these pH trends indicate that CIF changes with pH might depend on various factors, such as protonation equilibria involving TC^•+^, the reactions of transformation intermediates of the TCs with superoxide radical anion to form transformation products or leading to reformation of the parent compound, or with DOM, yielding addition of the oxidized TCs to DOM. The latter reaction, which was suggested to occur for aniline in an oxidative aqueous environment,^87^ possibly affects in particular the transformation of 4MA, whose CIF exhibits a very distinct pH dependence.

Comparing Figure 2a,c and b,d suggests that CIF tends to be lower for SRFA compared to PLFA. A direct comparison is provided in Figure 2f based on the normalized reaction rate constants *k*_TC,pH,norm,SRFA_^(2)^/*k*_TC,pH,norm,PLFA_^(2)^. For most TCs, this ratio is smaller than unity for any pH value ≥7, the lowest values being observed at 8 ≤ pH ≤ 10. This can be interpreted as an enhanced inhibitory effect of SRFA in the latter pH range compared to PLFA. In general, a higher inhibitory effect of SRFA compared to PLFA is expected based on previous studies and the higher concentration of antioxidant moieties in SRFA.^34−36^ The fact that the minimum values in CIF ratios are found in this pH range, but not exactly at the same pH value for any TC, suggests that the inhibitory effect is related to the protonation equilibria of phenolic moieties in DOM and the radical intermediates of each TC (i.e., TC^•+^). These aspects will be discussed in more detail when addressing results from System 4 experiments (vide infra).

A possible explanation for the different behavior of 4MtA in systems with CBBP and 2AN at pH below ∼7–8 could be that due to the high reduction potential of excited triplet CBBP, secondary DOM-derived photo-oxidants are formed, which can transform the readily oxidizable 4MtA and would mask DOM-induced inhibition observed in the systems with 2AN.

### Transformation of TCs by Model Photosensitizers in the Presence of DOM as an Antioxidant (System 2)

**Figure 3:**
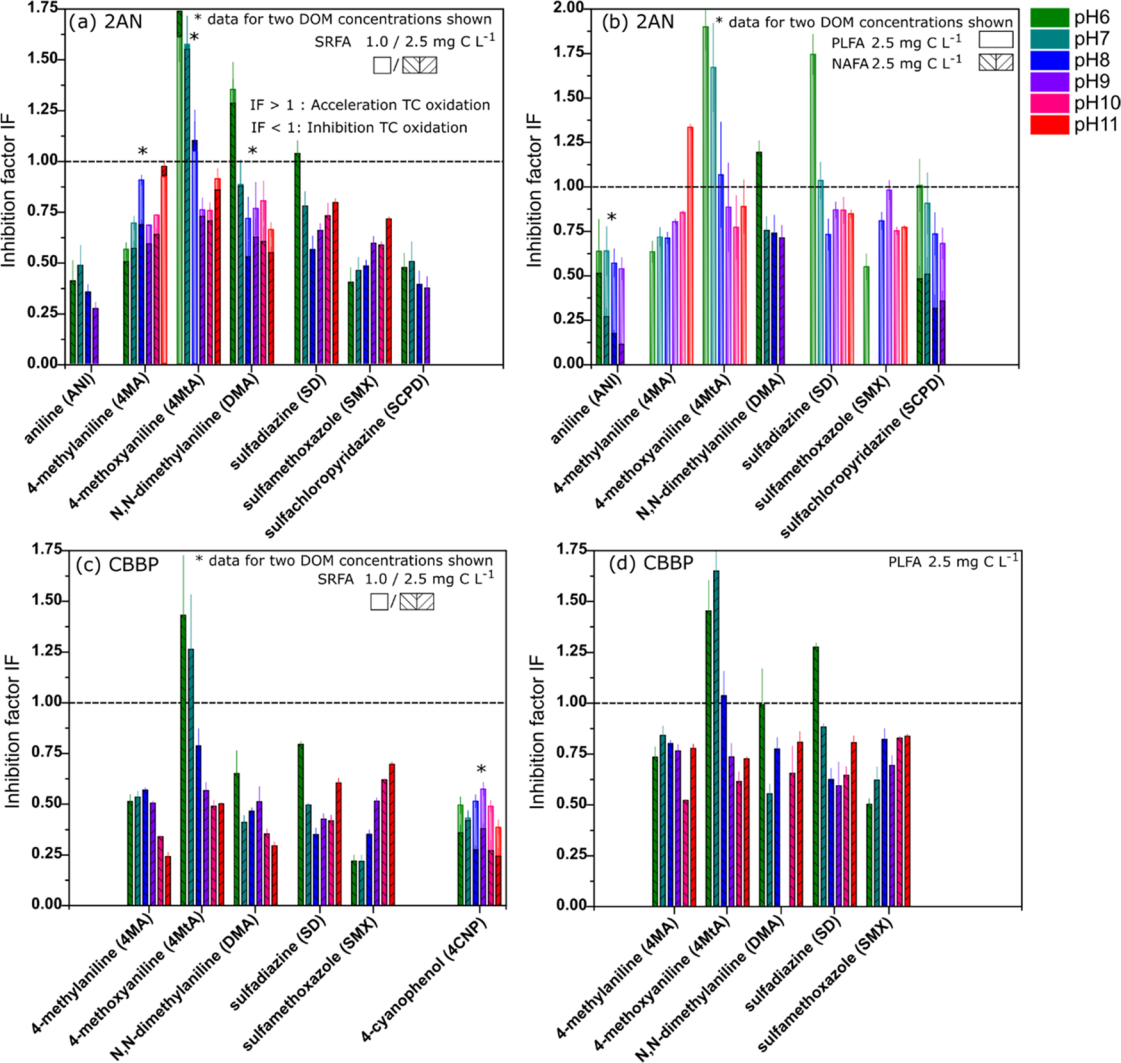
Inhibition factors (IF) for the transformation of target compounds (TCs; *x-*axis) photosensitized by 2-acetonaphthone (2AN) (a, b) and benzophenone-4-carboxylate (CBBP) (b–d) with the DOM isolates SRFA, PLFA, and NAFA in their role as natural antioxidants.

Figure 3 shows results for irradiations with model photosensitizers and DOM to assess the role of DOM as an antioxidant. Data are presented as inhibition factor (IF), which is the ratio of pseudo-first-order reaction rate constants with and without added DOM as an antioxidant (i.e., IF = *k*_TC,pH,Sens,AO_^(2)^/*k*_TC,pH,Sens_^(2)^). The corrected reaction rate constants used in the calculation of IF are provided in Figures S8–S16. For most of the studied systems across all TCs and pH values, there was a significant inhibitory effect (IF < 1). The determined IF values are like those reported previously at pH 8 for the same model photosensitizer, TC and DOM combinations.^33,34^ The inhibitory effect at 2.5 mg C L^–1^ DOM addition was stronger than at 1.0 mg C L^–1^ across the whole pH range. Higher IF values (i.e., weaker inhibitory effect) for microbially derived PLFA compared to terrestrially derived SRFA and NAFA confirm previously measured IF data at pH 8.^34^

Regarding the pH dependence of the inhibitory effect, four out of eight TCs (ANI, 4MtA, DMA, and SCPD) with 2AN as a photosensitizer showed a decreasing IF with increasing pH. For 4MA, SMX, and SD, there was either an increase in IF or no obvious trend with pH. The results for the latter TCs appear to not support the initial hypothesis that IF decreases (i.e., inhibition increases) with increasing pH, considering that the antioxidant capacity of DOMs (measured as electron-donating capacity, EDC) almost doubles by increasing the pH from 7 to 9.^43^ IF trends were similar for both 2AN and CBBP, except for 4MA. The data of 4-CNP, which show an almost constant inhibitory effect over the studied pH range (Figure 3c), could be explained by the high reduction potential (i.e., strongly oxidizing character) of the 4-cyanophenoxyl radical (1.12 V, see Table 1). This may cause a very efficient reduction of this radical by both undissociated and deprotonated phenolic antioxidant moieties of DOM, leading to re-formation of the parent compound and hence inhibited reaction. Furthermore, especially for 4MtA, but also for DMA and SD, some IF values >1 were observed in the lower pH range, meaning that for these TCs and these pH conditions an enhancement effect of DOM was dominant over a possible inhibitory effect. Analogous enhancements have been observed previously and attributed to the formation of oxidizing species resulting from the reaction of DOM with the primary oxidant, specifically, the triplet state of a model photosensitizer^33,34^ but also for the sulfate, radical which was studied separately.^88^ This enhancement effect could explain the unclear trends observed for System 2.

### Transformation of TCs by DOM as a Photosensitizer in the Presence of Phenol as an Antioxidant (System 3)

Experiments were performed using aniline as a TC, DOM isolates as photosensitizers, and phenol as an antioxidant in the pH range from 6 to 9. Their results are represented in terms of IF in Figure S17. While the inhibitory effect of phenol was almost absent at pH 6 (i.e., IF ≅ 1), it was significant at higher pH, with an increase in inhibition (i.e., decrease in IF) observed for PLFA and SRFA, and a pH-independent IF for NAFA. The effect of phenol addition on IF was more pronounced for PLFA than for SRFA or NAFA, which can be ascribed to the lower intrinsic phenol content of PLFA^32,50^ and concurs with the results of previous studies performed at pH near 8.^35,37^

### Transformation of TCs in the Presence of 2-Acetonaphthone as a Model Photosensitizer and Phenol as a Model Antioxidant (System 4)

For this part of the study, four anilines (ANI, 4MA, 4MtA, and DMA) and three sulfonamides (SD, SMX, and SCPD) were tested as TCs (Figure S18). The IF of all anilines decreased with increasing pH up to pH 9, in agreement with the basic hypothesis that the phenolate ion is a better inhibitor of these photosensitized reactions than the undissociated phenol. At pH 6, 4MA and DMA transformation was not inhibited, while for 4MtA, even an enhanced reaction (IF > 1) is observed at pH 6 and 7. At the highest pH of 11, a reversal in trend is observed for 4MA and 4MtA, i.e., IF is higher compared to pH 10. An extended discussion of the behavior of the anilines including transformation data is given in the next subsection. For sulfonamides, there is no obvious pH trend. SD exhibited the lowest IF at circumneutral pH as observed with natural antioxidants, while IF for SMX decreased with increasing pH.

For a rough comparison of the relative changes in IF with pH with phenol and the natural antioxidants SRFA, PLFA, and NAFA, the ratio of the corresponding IFs was calculated (i.e., IF_natural AO,pH_/IF_phenol,pH_, Figure S19). We note that such comparison needs to be interpreted carefully since antioxidant concentration and type are different. For 4MtA, SCPD, and SD, except at pH 7, the ratios are ∼1 over the whole pH range. This confirms the qualitative observation from above that IF values behave similarly with changing pH in the presence of either natural or model antioxidants. IF ratios for ANI, 4MA, and DMA increase with pH, indicating a relative increase of inhibition with increasing pH in systems with phenol as a model antioxidant.

### Correlation between the Inhibitory Effect and pH-Dependent Redox Potentials

**Figure 4:**
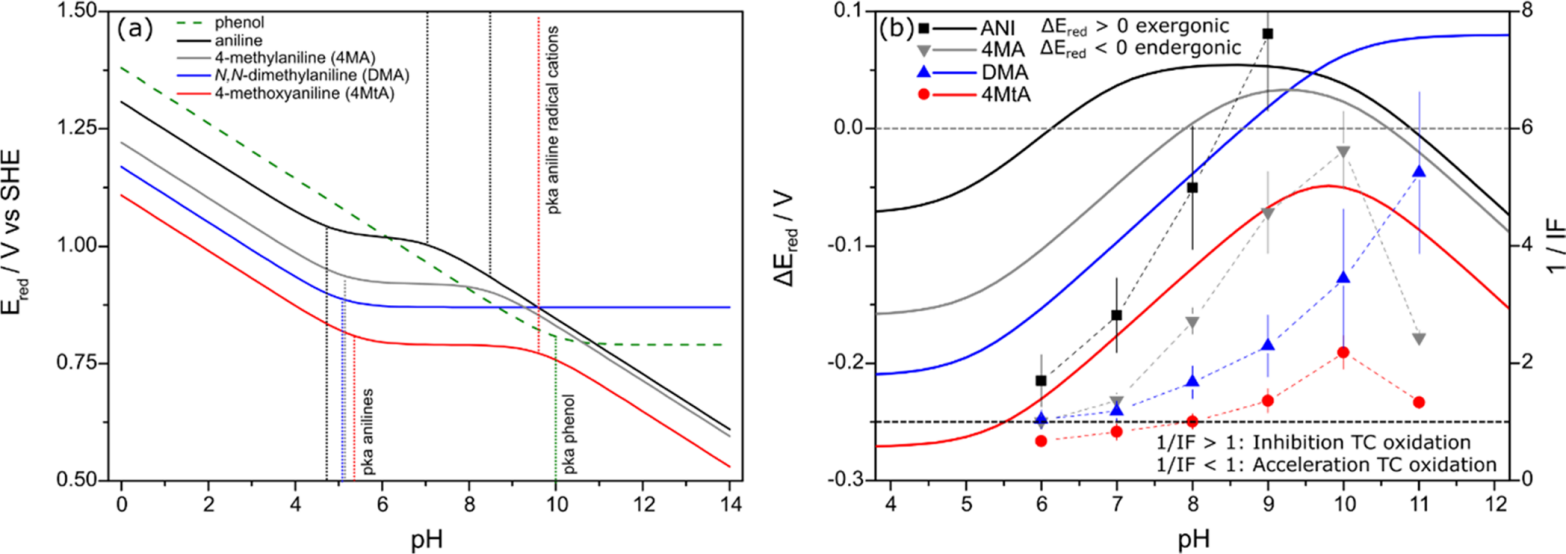
(a) One-electron reduction potentials *E*_red_ (V vs standard hydrogen electrode (SHE)) for phenoxyl and anilinyl radicals as a function of pH. Acid dissociation constants of the relevant species (see Table 1) are indicated by vertical dotted lines. (b) Difference in reduction potential Δ*E*_red_ of each anilinyl radical and phenoxyl radical for the pH range of 4–12 and inverse inhibition factors (1/IF) for anilines with 2AN as a model photosensitizer and phenol (10 μM) as a model antioxidant. Calculations for *E*_red_ and Δ*E*_red_ are available as Supporting Information .xlsx data file, with further consideration on the dependence of redox potentials of phenol and anilines, including redox equations provided in the SI, Text S6.

In systems consisting of both a model photosensitizer and a model antioxidant (System 4), pH-dependent redox potentials were used to rationalize the inhibitory effect across different TCs in the studied pH range. Both the model antioxidant phenol and the four aniline target compounds (ANI, 4MA, 4MtA, and DMA) have pH-dependent redox potentials, which are represented in Figure 4a. The one-electron reduction potential of the redox couple consisting of the phenoxyl radical (both protonated and neutral form) and the phenol (both molecular and deprotonated form) decreases linearly (slope of −0.059 V per pH unit) with increasing pH from 0 to 10, reaching a constant value of 0.79 V vs SHE above pH 10 at which reduction of the radical to the phenolate is not coupled to proton uptake.

For the anilinyl radicals (protonated and neutral forms are considered), the one-electron reduction potentials decrease in the same way as for phenol in the pH range from 0 to the p*K*_a_ values of the individual anilines (4.9–5.4), remain constant (i.e., pH independent) in the pH range between these values and the p*K*_a_ values of the aniline radical cations, and then decrease linearly with pH (slope of −0.059 V per pH unit) in the higher pH range. The only exception to this additional decrease in *E*_red_ with pH is DMA, which maintains a constant reduction potential even at high pH since its radical cation does not deprotonate over the studied pH range. Except for 4MtA, *E*_red_(pH) functions for anilinyl radicals, which for 0 < pH < 5, are lower than the corresponding function for the phenoxyl radical, cross at some pH value the *E*_red_(pH) function of the latter, meaning that the oxidation of phenol by the anilinyl radicals (cationic or neutral forms) becomes thermodynamically favorable above this pH. A second crossing of the reduction potentials of anilines and phenol occurs for ANI and 4MA at pH values well above the p*K*_a_ of phenol, and the oxidation of phenol by the corresponding anilinyl radicals becomes thermodynamically unfavorable above these pH values.

To better illustrate the thermodynamics of these redox reactions, Figure 4b shows the difference of reduction potential between each anilinyl and phenoxyl radicals (i.e., Δ*E*_red_ = *E*_red,anilinyl_ – *E*_red,phenoxyl_). The diagram also contains data on the inhibitory effect, expressed as inverse inhibition factor (1/IF: high 1/IF corresponds to high inhibitory effect) for the four anilines. The magnitude of the inhibitory effect clearly correlates with increasing Δ*E*_red_ in the order of ANI > 4MA > DMA > 4MtA and follows, for each of the anilines, the pH-dependence of Δ*E*_red_. It can be concluded that the inhibition is determined by the pH (and speciation)-dependent reduction potential difference between the phenoxyl/phenol and the anilinyl/aniline couples. Interestingly, inhibition (1/IF > 1) of aniline oxidation was observed even for thermodynamically unfavorable conditions, i.e., negative Δ*E*_red_. For Δ*E*_red_ < 0 V, the reaction rate constant for the reduction should be smaller and eventually become insignificant compared to conditions with positive Δ*E*_red_. Simultaneously, for Δ*E*_red_ < 0 V, the back reaction, i.e., the oxidation of the aniline via the phenoxyl radical is expected to be faster than the forward reaction.

To explain the observed IF under these conditions, scavenging of the phenoxyl radical, which outcompetes the back reaction, must be assumed. Possible scavengers of the phenoxyl radical include the phenoxyl radical itself, superoxide, and phenol,^89^ the latter reaction leading to radical adducts.^90^ The aforementioned thermodynamic considerations also offer arguments to explain the reaction enhancement (IF > 1) observed for 4MtA at pH 6 and 7 (see the preceding subsection). Phenoxyl radicals, which may be formed by oxidation of phenol by the excited triplet state of 2-AN, may cause an effective oxidation of 4MtA, since this reaction is the most favorable among the ones considered (see Figure 4b). In turn, the anilinyl radicals of 4MtA cannot be reduced by phenol. As a result, the oxidation of 4MtA by phenoxyl radicals prevails, causing an enhancement of the photosensitized transformation of 4MtA.

The Δ*E*_red_ vs pH trends presented in Figure 4b, which exhibit maxima in correspondence of 1/IF maxima, can explain qualitatively the occurrence of the minima in IF (corresponding to maxima in 1/IF) observed in several cases for System 2 data (see Figure 3). This observation can be extended by analogy to the minima in CIF observed for System 1 data (see Figure 2). The fact that, for DOM acting as an antioxidant, the minima in IF or CIF occur at lower pH than for phenol might be due to the lower p*K*_a_ of phenolic moieties of DOM^32^ compared to phenol.

For sulfonamides, predictions about IF based on Δ*E*_red_ can only be made at the level of guesses due to the missing knowledge on the reduction potentials of their radicals and the complex speciation behavior of the latter.^82^ Radicals of the anilinium type would have a higher one-electron reduction potential (e.g., ∼1.3 V vs SHE for SD)^91^ and are also expected to have lower p*K*_a_^*^ values (e.g., ∼6.3 estimated for SD using quantum chemical computations)^82^ than the corresponding radicals of the anilines studied here. The higher reduction potentials can explain generally the lower IF values (more efficient inhibition) observed, especially in the lower pH range, for the sulfonamides compared to the anilines. Lower p*K*_a_^*^ values would explain why the IF minima and onsets to increasing IF would occur at lower pH for the sulfonamides compared to the anilines.

## Environmental Implications

This study presents the first comprehensive collection of kinetic data on the pH dependence of the photosensitized transformation of aromatic amines under conditions relevant to sunlit surface waters. Pseudo-first-order rate constants for aromatic amines photosensitized by PLFA and SRFA, as surrogates of dissolved organic matter present in surface waters, varied depending on the specific target compound by a factor of up to ∼7 in the 6–11 pH range. For the realistic situation encountered in most freshwaters buffered by bicarbonate/carbonate, pH varies between 7 and 9, and the pH-induced variability of pseudo-first-order rate constants reduces significantly for several of the studied compounds. However, at the smaller range of environmentally occurring pH, the maximum observed variability factor of ∼5 remains high. Therefore, to accurately predict the fate of aromatic amines in surface waters, investigations on the pH dependence of their photosensitized transformation may be required.

It is generally assumed that the transformation kinetics and abatement of contaminants in the aquatic environment can be described in terms of their speciation using corresponding rate constants (independent of the water matrix composition) for each species and, when applicable, the steady-state concentration of aquatic reactive species (such as ^3^DOM*, ^1^O_2_, or the hydroxyl radical). However, this approach has limitations, and the present study has highlighted, for the studied aromatic amines, the existence of a pH-dependent photosensitized transformation kinetics beyond a simple speciation effect.

The hypothesis of an increased inhibitory effect of DOM on photosensitized transformation of aromatic amines at higher pH could not be confirmed for several studied compounds. A possible explanation for this failure is the important increase in the efficiency of photosensitized transformation with increasing pH, observed for several compounds. Moreover, other not yet well understood compound-specific effects, such as protonation equilibria, lifetimes, and reactions (possibly addition to and reduction by superoxide radical anion, and addition to DOM) of the radicals derived from the oxidation of the compounds, might affect transformation rates of these compounds. Beyond oxidations induced by excited triplet states, the inhibitory effect of DOM may also occur for the radical-induced reactions of organic contaminants, as recently shown for the sulfate radical^88^ produced by photolysis of persulfate. For the latter system, a clearer response of the inhibitory effect of DOM with increasing pH is expected due to the absence of significant superoxide sources that would compete with DOM-induced inhibition.

## Supporting Information Available

*E*_red_ and Δ*E*_red_ calculations (XLSX)
Chemicals used, preparation of stock solutions, analytical details, discussing direct phototransformation of target compounds, effects of changes in absorption spectra, role of singlet oxygen and superoxide, relationships of redox potentials of phenol and anilines with pH (Texts S1–S6); HPLC analysis parameters, direct phototransformation rates, measured and corrected photosensitized transformation rates, absorption coefficients of photosensitizers, including for DOMs, and light attenuation factors of experimental solutions (Tables S1–S14); and UV–vis absorption spectra of photosensitizers, DOMs, and target compounds, depletion kinetics of target compounds and phenol, and phototransformation rates with model sensitizers in the presence of model and natural antioxidants (Figures S1–S19) (PDF)
