# Supplementary material for: Effect of Solution pH on the Dual Role of Dissolved Organic Matter in Sensitized Pollutant Photooxidation
Source: Environ Sci Technol. 2021 Oct 29;55(22):15110–22. doi: 10.1021/acs.est.1c03301 (PMC8735754; doi:10.1021/acs.est.1c03301)
Supplement: Supplementary file 2 — es1c03301_si_002.pdf [file es1c03301_si_002.pdf]

Supporting Information

Effect of solution pH on the dual role of dissolved organic matter in sensitized pollutant photooxidation

Jannis Wenk[*,1,2,3], Cornelia Graf[2,4], Michael Aeschbacher[2], Michael Sander[2], Silvio Canonica[*,1]

[1]Eawag, Swiss Federal Institute of Aquatic Science and Technology,
CH-8600, Dübendorf, Switzerland

[2]Institute of Biogeochemistry and Pollutant Dynamics, ETH Zürich,
CH-8092, Zürich, Switzerland

[3]Department of Chemical Engineering and Water Innovation & Research Centre (WIRC),
University of Bath, Claverton Down, Bath BA2 7AY, United Kingdom

[4]Present address: INFRAS Research and Consulting, CH-3012, Berne, Switzerland

*Corresponding authors:
E-mail: j.h.wenk@bath.ac.uk
Phone: + 44-1225-383246

E-mail: silvio.canonica@eawag.ch
Phone: +41-58-765-5453
Fax: +41-58-765-5210

<u>Summary:</u>

This file has **40 pages** and **29 references** and contains

6 Texts specifying chemicals used, preparation of stock solutions, analytical details, discussing direct phototransformation of target compounds, effects of changes in absorption spectra, role of singlet oxygen and superoxide, relationships of redox potentials of phenol and anilines with pH.

14 Tables showing HPLC analysis parameters, direct phototransformation rates, measured and corrected photosensitized transformation rates, absorption coefficients of photosensitizers, including for DOMs, and light attenuation factors of experimental solutions.

19 Figures showing UV-Vis absorption spectra of photosensitizers, DOMs, and target compounds, depletion kinetics of target compounds and phenol, and phototransformation rates with model sensitizers in the presence of model and natural antioxidants.

Text S1: Chemicals

Target compounds (TCs). 4-cyanophenol (4CNP) (puriss. p.a. ≥ 97%), *N,N*-dimethylaniline (DMA) (puriss. p.a. ≥ 99.5 %) and 4-methylaniline (4MA) (puriss. p.a. ≥ 99%) were purchased from Fluka (St. Louis, MO, USA). 4-methoxyaniline (MtA) (puriss. p.a. ≥ 99%), sulfadiazine (SD) (≥99%), sulfachloropyridazine (SCPD) and sulfamethoxazole (SMX) were purchased from Sigma-Aldrich (St. Louis, MO, USA).

Model photosensitizers. 2-acetonaphthone (2AN) (puriss. p.a. ≥ 99%) and 4-carboxybenzophenone (CBBP) (puriss. p.a. ≥ 99%) were from Sigma-Aldrich.

Humic substances and model antioxidant (AO). All humic and fulvic acids were purchased from the International Humic Substances Society (IHSS, St. Paul, MN, USA): Pony Lake fulvic acid reference (1R109F, PLFA), Suwannee River fulvic acid standard II (2S101F, SRFA), Nordic aquatic fulvic acid reference (1R105F, NAFA). Phenol (puriss. p.a. ≥ 99.5%) was purchased from Fluka.

Buffer solutions. For photochemical experiments: ortho-phosphoric acid (puriss. p.a. ≥ 85%) was from Fluka, sodium dihydrogen phosphate monohydrate (puriss. p.a. ≥ 99%-102%), disodium hydrogen phosphate dihydrate (puriss. p.a. ≥ 99.5%) and trisodium phosphate dodecahydrate (puriss. p.a. ≥ 97.5-102%) were from Merck (Darmstadt, Germany).

Stock solutions. Phosphate buffer stock solutions (50 mM) and unbuffered aqueous stock solutions (1 mM) of TCs (Table 1) were stored at 4°C and renewed at least monthly. Fresh DOM stock solutions (pH 8, 5 mM phosphate buffer, 100 – 200 mg C L^{-1}) were prepared as needed. Stock solution of 2-acetonaphthone (2AN, 100 mM) was prepared in pure methanol[1].

Text S2: Analytical methods

Measurements of pH were carried out at room temperature on a Metrohm pH meter model 632 (Herisau, Switzerland) calibrated daily prior to the first sample measurements. Absorption spectroscopy was conducted using a Cary 100 UV-Vis (Agilent, Technologies) equipped with 10- or 20-mm path length quartz glass cuvettes (Hellma). The spectrum of buffered water at the appropriate pH was subtracted from each sample spectrum. Solution pH values and UV-Vis absorption were determined at room temperature. HPLC analyses were performed on a Dionex UltiMate® 3000 or an Agilent 1100 Series LC system equipped with UV-Vis absorbance and fluorescence detectors. A table of HPLC methods for all TCs is given in Table S1. Further details on HPLC equipment and methods are provided elsewhere.[1, 2]

Table S1. Isocratic HPLC analysis parameters

Target compound	Composition of mobile phase [%]		Retention time [min]	Absorption wavelength [nm]	Excitation/ Emission wavelength [nm] [3]	eluent buffer pH
	Acetonitrile	Buffer [10 mM]				
4-cyanophenol	30	70	1.9-2.5 [1,2]	220	**275/310**	2
4-methoxyaniline	20	80	2.8 [1,2]	220	**236/362**	7
4-methylaniline	40	60	2.4 [1]	220	**233/346**	7
aniline	50-10	50-90	2.1-8.1 [1]	220	**236/341**	7
N,N-dimethylaniline	50	50	4.2 [2]	220	**251/360**	7
phenol	30-10	70-90	3.4-9.1 [1]	220	**275/310**	2/7 [4]
sulfachloropyridazine	30-15	70-85	3.1-8.6	270	**-**	2
sulfadiazine	30-10	70-90	1.9-4.4 [2]	270	**-**	2
sulfamethoxazole	30-20	70-80	3.4-6.6 [2]	270	**-**	2

[1]: COSMOSIL Packed Column 5C18-MS-II, 100 x 3 mm, at a flow rate of 0.6 mL min^{-1}

[2]: NUCLEOSIL Packed Column 100-5 C18, 125 X 4 mm, at a flow rate of 1 mL min^{-1}, reverse-phase columns purchased from Macherey-Nagel (Düren, Germany)

[3]: Preferred detection method indicated in bold

[4]: analysed at pH 7 in samples containing anilines

Text S3: Direct phototransformation

To account for direct photoreactivity for data correction and to ensure TC stability during the irradiation period, particularly at more alkaline pH, experiments were conducted with single TCs or the model antioxidant phenol (section 3) in the absence of photosensitizers (Tables S2 and S3). UV spectra of TCs at different pH were recorded (Figure S1 and S2). Little or no direct phototransformation for most TCs and phenol was observed which was expected when comparing irradiation conditions with UV absorption spectra of TCs at different pH. Significant pH-dependent changes of direct phototransformation rates were observed for sulfadiazine. For aniline, 4-methoxyaniline and 4-methylaniline transformation kinetics at irradiation times > 10 min deviated from pseudo-first order kinetics indicating autocatalytic oxidative polymerization in pure buffered water [3-5] (Figures S3 and S4), proceeding faster with increasing pH. Aniline autocatalysis was unimportant for experiments with model photosensitizers which were finished after 5 minutes. Transformation kinetics for the three anilines using natural photosensitizers showed no autocatalytic behavior. No mutual effect or significant changes of direct phototransformation rates of individual TCs in presence of phenol without photosensitizers was observed. However, phenol depleted rapidly at higher pH in combination with 4-methylaniline, 4-methoxyaniline, DMA, SD and SMX (Table S3). The implications of changes in absorption spectra with pH on indirect photochemical reactions are discussed in Text S4

Table S2. Measured pseudo-first-order direct phototransformation rate constants (s^{-1}) for target compounds

Substance	Irradiation time (min)	Rate constant (s^{-1}) ± 95% confidence interval					
		pH6	pH7	pH8	pH9	pH10	pH11
aniline (Ani)	90	n.d.	n.d.	*	*		
4-methylaniline (4MA)	60	n.d.	n.d.	*	*	*	*
4-methoxyaniline (MtA)	60	$(8.0±2.0)×10^{-6}$	$(3.6±0.6)×10^{-5}$	$(4.5±0.8)×10^{-5}$	*	*	*
N,N-dimethylaniline (DMA)	60	n.d.	$(1.9±0.2)×10^{-5}$	$(2.5±0.7)×10^{-5}$	$(2.5±0.5)×10^{-5}$	$(1.7±0.3)×10^{-5}$	$(2.3±0.3)×10^{-5}$
sulfamethoxazole (SMX)	60	$(6.6±1.1)×10^{-6}$	$(4.2±0.2)×10^{-6}$	$(2.8±0.8)×10^{-6}$	$(2.7±1.1)×10^{-6}$	$(7.6±1.4)×10^{-6}$	$(2.7±0.6)×10^{-6}$
sulfachloropyridazine (SCPD)	60			$(1.2±0.1)×10^{-4}$			
sulfadiazine (SD)	60	$(1.0±0.1)×10^{-5}$	$(2.1±0.1)×10^{-5}$	$(2.9±0.1)×10^{-5}$	$(3.6±0.3)×10^{-5}$	$(4.7±0.5)×10^{-5}$	n.d.
4-cyanophenol (4CNP)	5	n.d.	$(1.5±0.2)×10^{-4}$	$(2.3±0.5)×10^{-4}$	n.d.	n.d.	n.d.
phenol	60	n.d.	n.d.	n.d.	n.d.	n.d.	n.d.

n.d.: depletion not detected above level of uncertainty.

*: deviation from pseudo-first order transformation kinetics / autocatalytic transformation.

Table S3. Measured pseudo-first order direct phototransformation rate constants (s^{-1}) for target compounds in presence of phenol

Substance	Irradiation time (min)	Rate constant (s^{-1}) ± 95% confidence interval					
		pH6	pH7	pH8	pH9	pH10	pH11
aniline (Ani)	90	$(5.3±1.8)×10^{-6}$	$(2.6±2.0)×10^{-6}$	$(3.8±1.2)×10^{-6}$	$(4.5±1.8)×10^{-6}$		
4-methylaniline (4MA)	60	n.d.	n.d.	n.d.	n.d.	n.d.	*/**
4-methoxyaniline (MtA)	60	$(1.1±0.2)×10^{-5}$	$(2.2±0.3)×10^{-5}$	$(6.8±0.5)×10^{-5}$	*/**	*/**	*/**
N,N-dimethylaniline (DMA)	60	n.d.	$(1.2±0.3)×10^{-5}$	$(2.0±0.6)×10^{-5}$		$(6.9±0.4)×10^{-5}$ **	$(9.3±0.6)×10^{-5}$ **
sulfamethoxazole (SMX)	60	n.d.	n.d.	n.d.	n.d. **	n.d. **	n.d. **
sulfachloropyridazine (SCPD)	60						
sulfadiazine (SD)	60	$(1.4±0.2)×10^{-5}$	$(1.0±0.1)×10^{-5}$	n.d. **	n.d. **	n.d. **	n.d. **
4-cyanophenol (4CNP)	5						

n.d.: depletion not detected above level of uncertainty.
*: deviation from pseudo-first order transformation kinetics / autocatalytic transformation.
**: phenol depletion

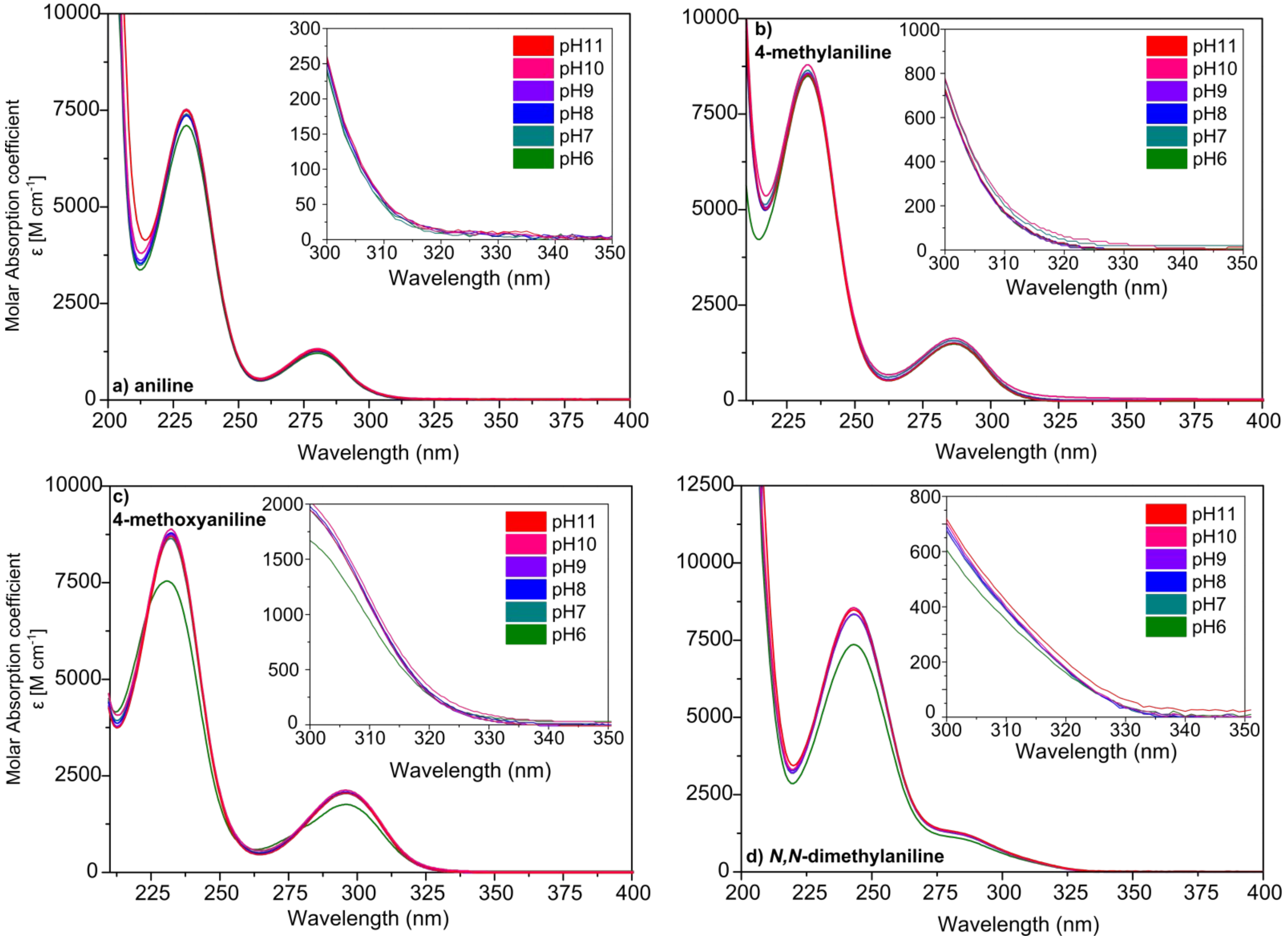

Figure S1. UV absorption spectra of target compounds (a) aniline, (b) methylaniline, (c) 4-methoxyaniline and (d) *N,N*-dimethylaniline at pH 6 – 11.

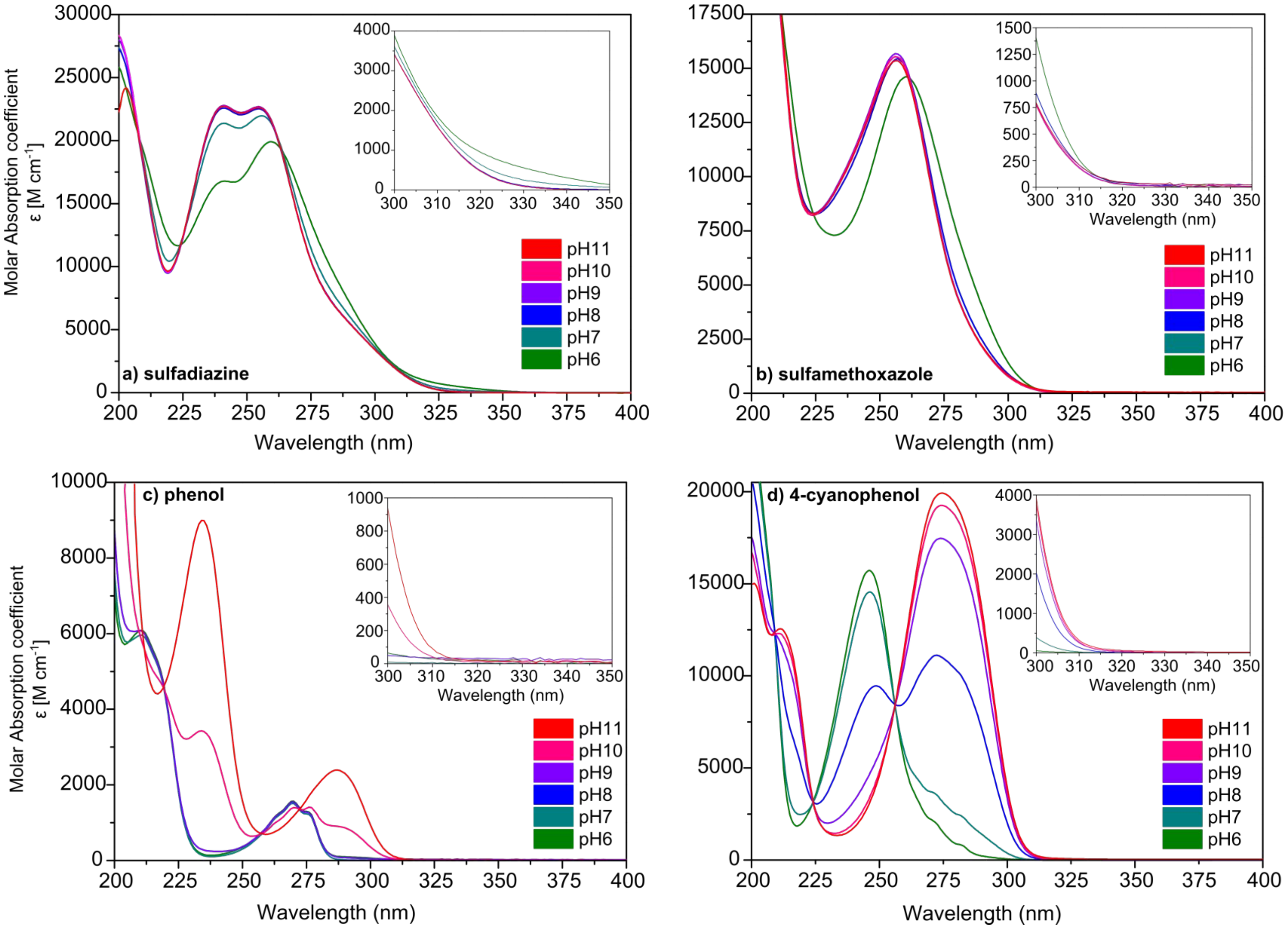

Figure S2. UV absorption spectra of target compounds (a) sulfadiazine, (b) sulfamethoxazole and phenolic compounds (c) phenol and (d) 4-cyanophenol at pH 6 – 11

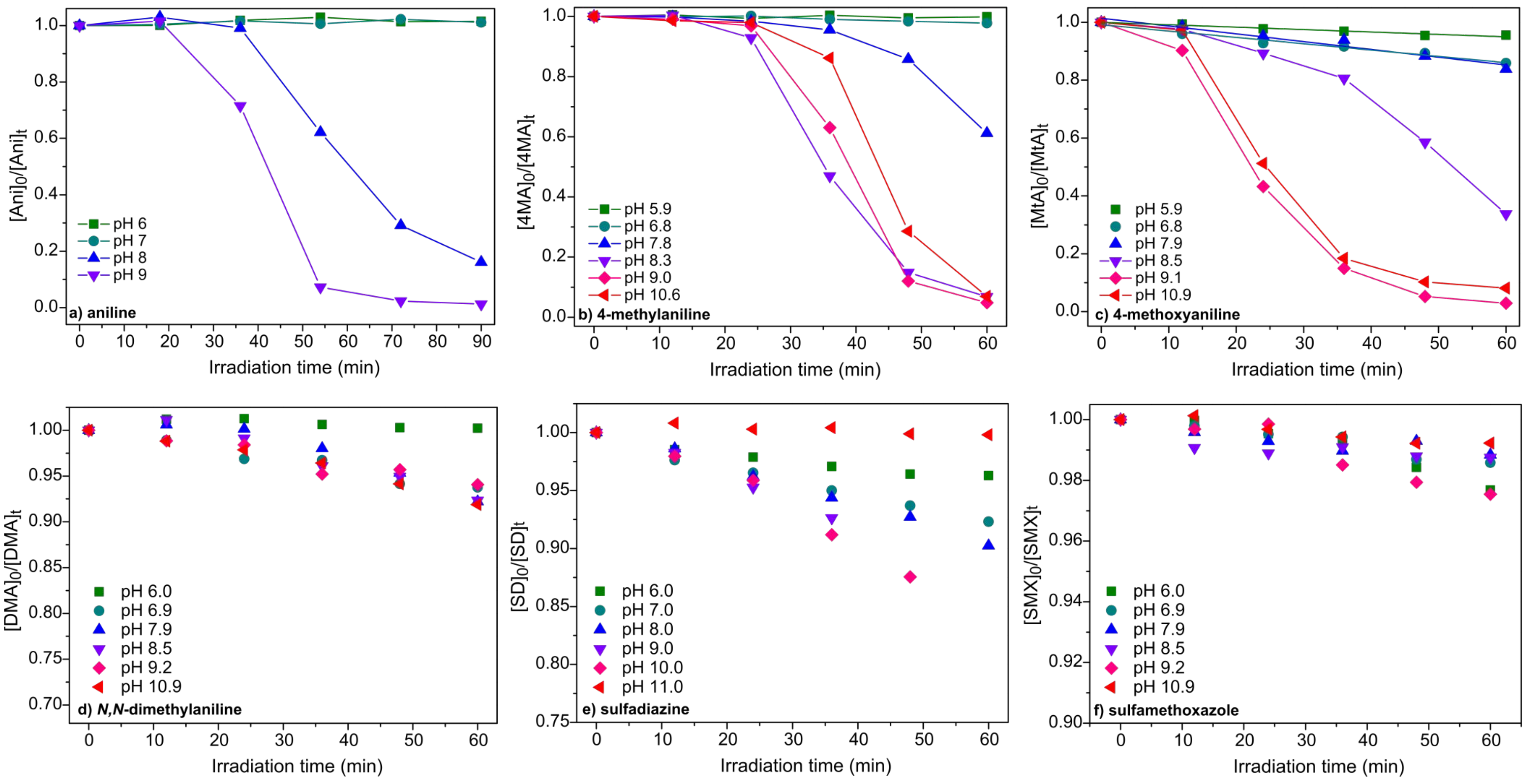

Figure S3. Normalized depletion kinetics of anilines and sulfonamides (all 5µM initial concentration): (a) aniline (Ani), (b) 4-methylaniline (4MA), (c) 4-methoxyaniline (MtA) and (d) *N,N*-dimethylaniline (e) sulfadiazine (SD) and (f) sulfamethoxazole (SMX) irradiated in 10mM phosphate buffered ultrapure water at pH 6 – 11.

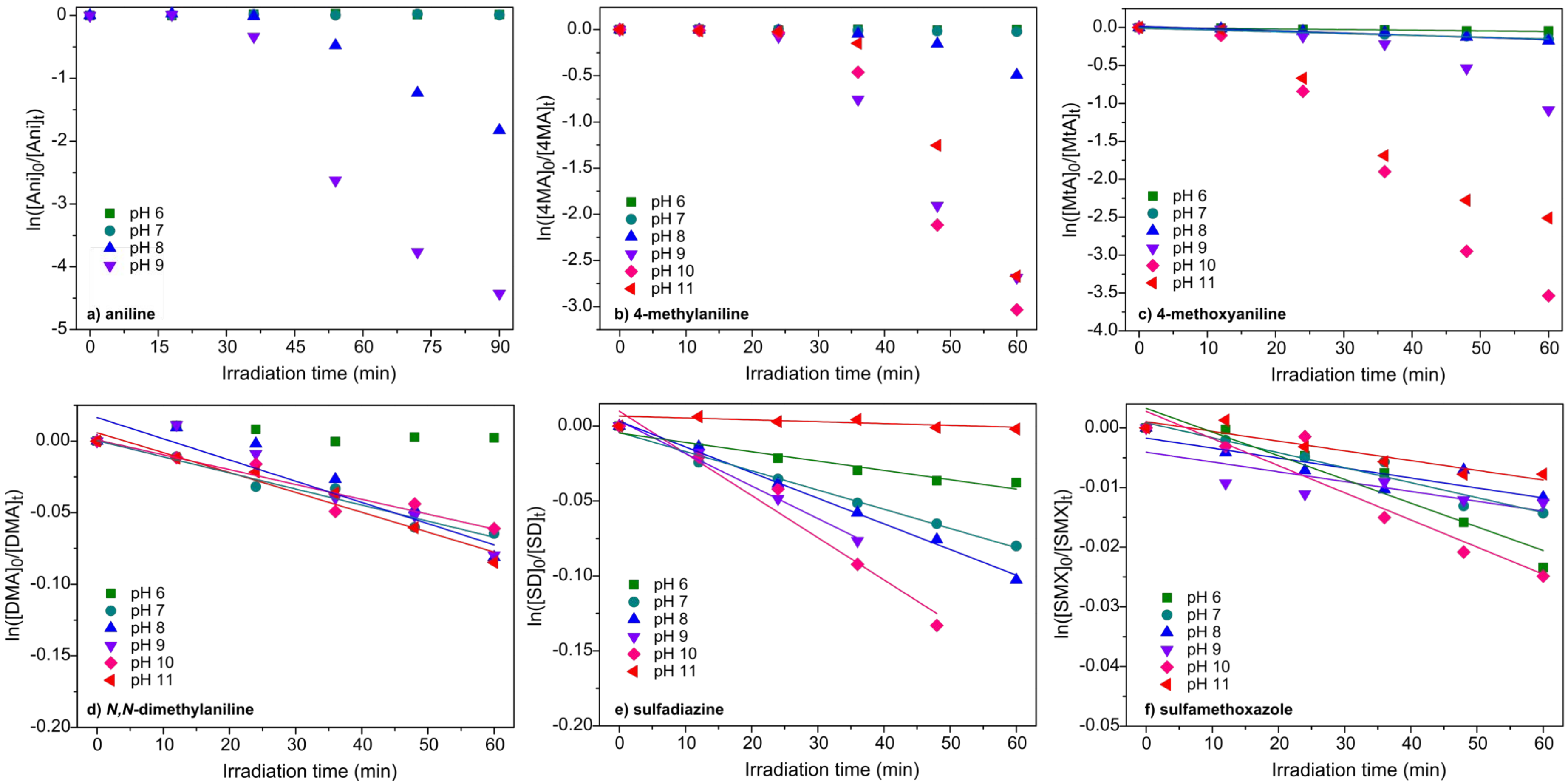

Figure S4. Logarithmic depletion kinetics of anilines and sulfonamides (all 5µM initial concentration): (a) aniline (Ani), (b) 4-methylaniline (4MA), (c) 4-methoxyaniline (MtA) and (d) *N,N*-dimethylaniline (e) sulfadiazine (SD) and (f) sulfamethoxazole (SMX) irradiated in 10mM phosphate buffered ultrapure water at pH 6 – 11.

Text S4. Absorption spectra

Compound specific alteration of absorption spectra of photosensitizers with pH may change the number of photons available to induce photoexcitation and thus lead to moderate over- or underestimation of indirect phototransformation kinetics trends of TCs when comparing across different pH and photosensitizers. This effect has been accounted for in the data correction and is in addition to changes in light-screening. For model photosensitizers the absorption spectra remained unchanged within the tested range of pH 6 – 11 (Figure S5/Table S4). Light absorption of DOMs between 300 and 400 nm increased steadily by a total of about 5% per pH unit (Figure S3/Table S4), in agreement with previous studies.[6, 7] This translates in an increase of absorption of 24% for PLFA and 25% for SRFA over the pH range, respectively (weighted for specific transmission and photon fluxes).[2] Beyond changes in absorption it is difficult to predict effects of pH on photophysical processes such as intersystem crossing (ISC) that influence $^3DOM^*$ quantum yields or $^3DOM^*$ reduction potentials, given the absence of studies addressing these points. However, DOM fluorescence intensity increases with pH[8] indicating that pH affects ISC of photoexcited DOM. We expect that triplet-state generation for 2AN and CBBP was unaffected by solution pH.

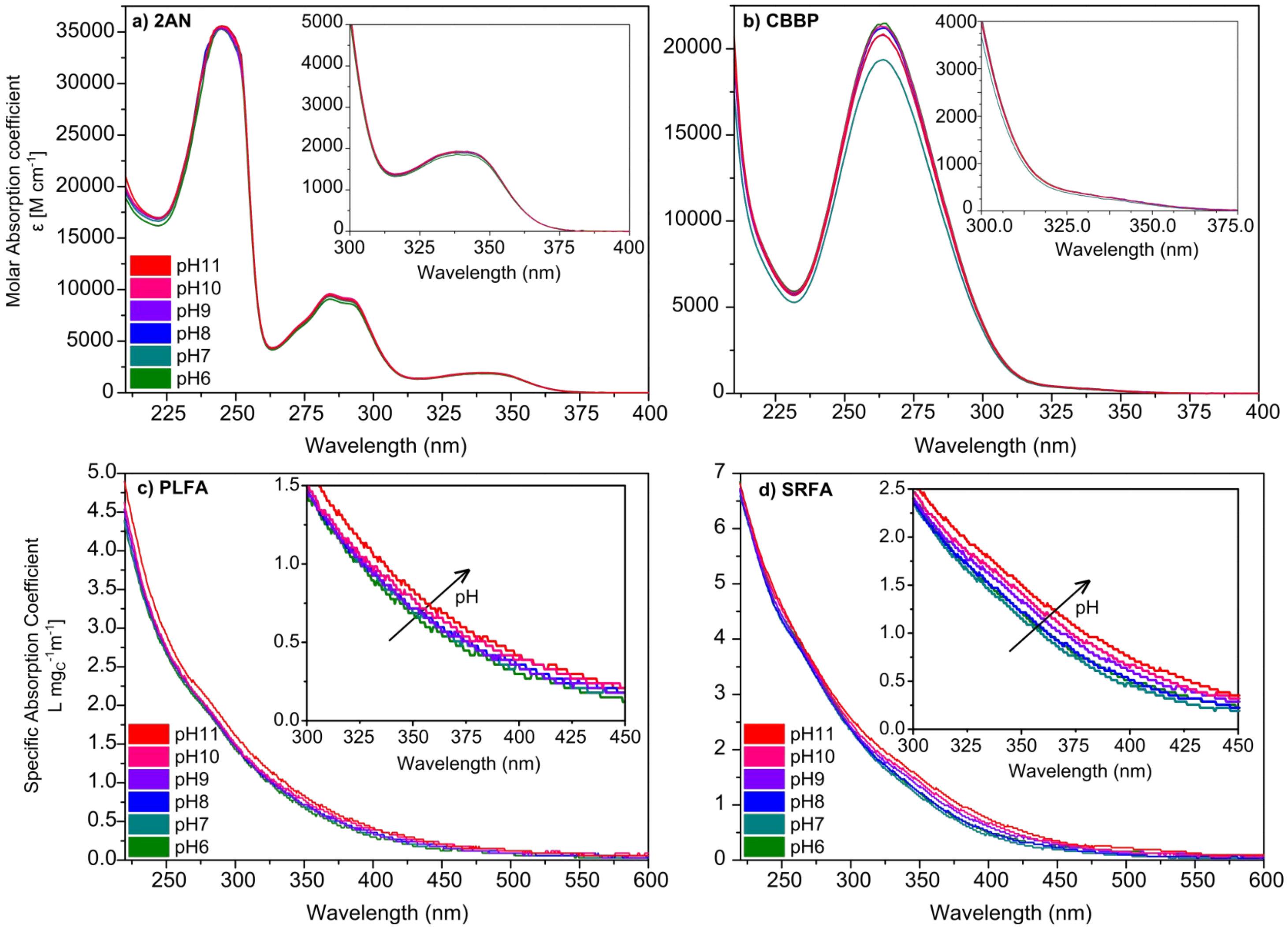

Figure S5. UV-Vis absorption spectra of photosensitizers (a) 2-acetonaphthone (2AN), (b) 4-carboxybenzophenone (CBBP), (c) Pony lake fulvic acid (PLFA) and (d) Suwannee river fulvic acid (SRFA) at pH 6 – 11.

Table S4. Light-absorption of photosensitizers at relevant wavelength: Specific absorption coefficients for SRFA/PLFA [L mg_C^{-1} m^{-1}] at pH 6 – 10 and molar absorption coefficients ε [M cm^{-1}] 2AN/CBBP

Wavelength (nm)	Specific absorption coefficients 334nm [L mg_C^{-1} m^{-1}]		Specific absorption coefficients 366nm [L mg_C^{-1} m^{-1}]	
Photosensitizer	SRFA	PLFA	SRFA	PLFA
pH				
6	1.47	0.87	0.86	0.54
7	1.54	0.90	0.92	0.56
8	1.54	0.93	0.93	0.57
9	1.63	0.94	1.02	0.58
10	1.63	0.96	1.12	0.63
11	1.82	1.04	1.22	0.66
Photosensitizer	2AN	CBBP	2AN	CBBP
	1864±30*	315±6*	276±4*	32±5

*Standard deviation of six measurements at pH 6-11.

Text S5. Superoxide and singlet oxygen

The superoxide radical anion might affect the pH dependence of the transformation rate constants of target compounds. Superoxide is a redox-active species and formed during excited triplet-induced oxidations of the type studied here.[9, 10] Superoxide could react with oxidation intermediates of some TCs, either by addition, leading to hydroperoxide-type products, or as a reductant, leading to reformation of the TC.[11, 12] The first reaction would accelerate, while the second reaction would slow down target compound transformation. A possible increase or decrease of phototransformation rates (depending on the relative importance of the mentioned reactions) with increasing pH would result from the decreasing dismutation rates, and consequently increasing lifetimes, of the superoxide radical anion with rising pH for pH > 5.[13, 14]

The potential contribution of singlet oxygen ($^1O_2$) on the observed kinetic trends also needs to be considered. Singlet oxygen forms by the reaction of excited triplet states with dissolved molecular oxygen (O_2).[15] We consider that the O_2 concentration in our experimental setup was constant as O_2 solubility is not directly affected by pH but rather by temperature and dissolved ion concentration,[16, 17] to which H^+ and OH^- do not contribute significantly in the studied pH range. Changes in the production rate of $^1O_2$ with pH are generally sensitizer-dependent and predictable if a sensitizer exhibits pH-induced shifts of its absorption spectra or has non-chromophore functional groups with pK_a relevant to the considered pH range,[18] neither of which applies to 2AN and CBBP (Figure S1). For $^3DOM^*$-sensitized $^1O_2$ formation, pH possibly has a modest effect. For example, $^1O_2$ quantum yields for Suwannee River DOM have been found to gradually decrease by approximately 25% between pH 4 and pH 10, while it was noticed that this trend is not predictive for other types of DOM.[19] The reactivity of the studied TCs or related compounds with $^1O_2$ is known through available second-order rate constants and indirect estimations. Isotope fractionation

experiments provided indirect evidence that $^1O_2$ does not contribute significantly to the oxidation of ANI, 4MA, 4MtA with methylene-blue as a photosensitizer.[20] The second-order rate constant for *N,N*-DMA with singlet oxygen is in the range of 10^8 M^{-1} s^{-1}.[21] More recently, the second-order rate constant for the reaction of $^1O_2$ with DMA related *N,N*-dimethyl-4-cyanoaniline (DMABN) was determined as < 1.9×10^7 M^{-1} s^{-1}, and the contribution of $^1O_2$ to the transformation of DMABN sensitized by PLFA was estimated as < 5%.[22] Second-order rate constants for fenamates (amine-derivatives) are in the range of (1.3 – 2.8) × 10^7 M^{-1} s^{-1}.[23] The contribution $^1O_2$ to the phototransformation SD and SCPD has been found insignificant.[24] Moreover, second-order rate constants for the reaction with $^1O_2$ have been evaluated for the following compounds: the anionic species of SMX (< 2 × 10^4 M^{-1} s^{-1})[25], the molecular ((2.4 ± 4.4) × 10^5 M^{-1} s^{-1}) and anionic form ((6.15 ± 0.27) × 10^6 M^{-1} s^{-1}) of 4-CNP[26], and TRP ((2.2 ± 0.4) × 10^7 M^{-1} s^{-1}or (3.43 ± 0.02 × 10^7 M^{-1} s^{-1}).[27, 28] Given above considerations and the mostly low reactivities of the studied TCs with $^1O_2$, we expect that $^1O_2$ had at most a minor effect on the observed pH trends of rate constants in this study.

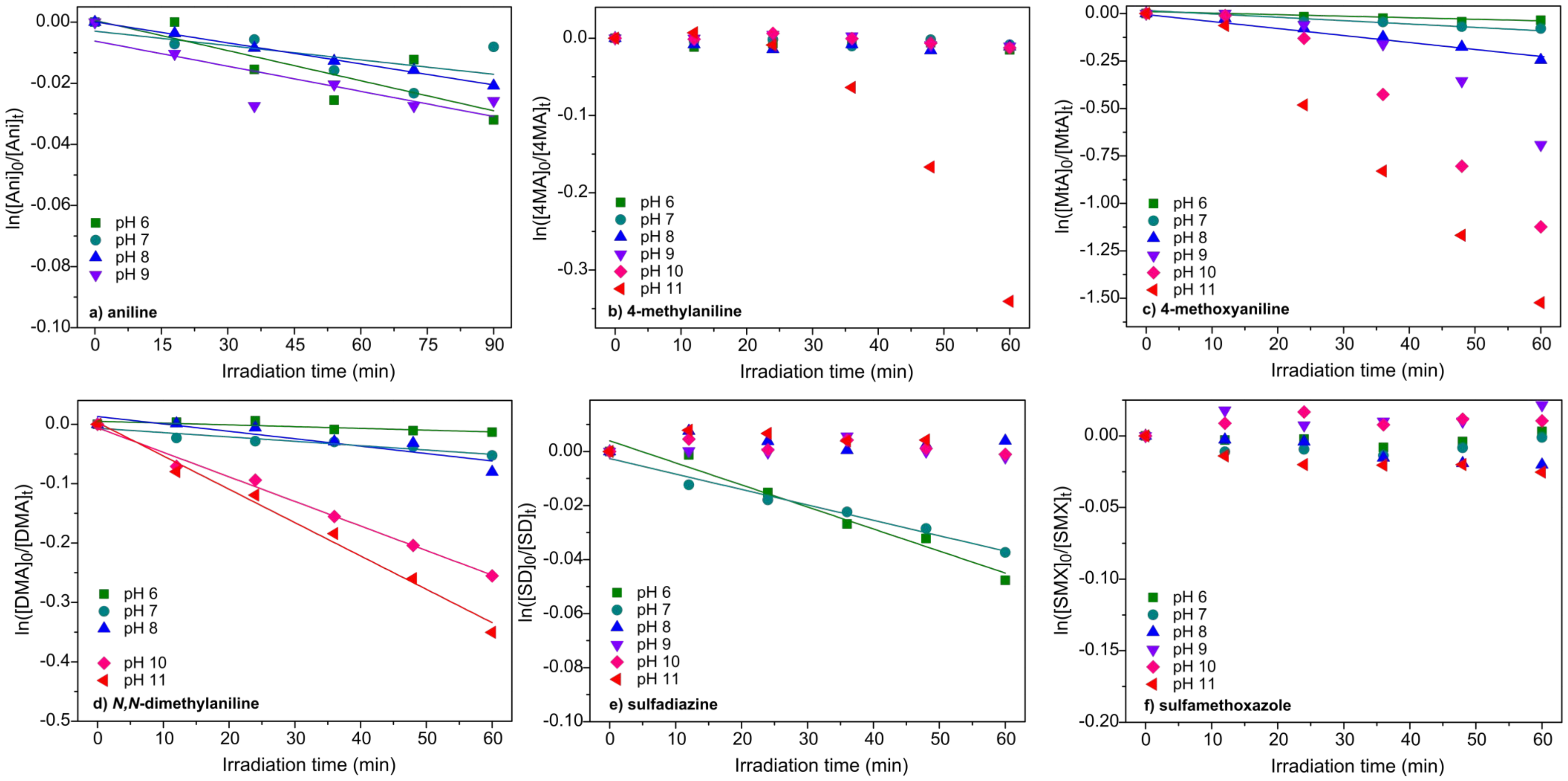

Figure S6. Logarithmic depletion kinetics of anilines and sulfonamides (all 5µM initial concentration) in presence of 10 µM phenol (a) aniline (Ani), (b) 4-methylaniline (4MA), (c) 4-methoxyaniline (MtA) and (d) *N,N*-dimethylaniline (e) sulfadiazine (SD) and (f) sulfamethoxazole (SMX) irradiated in 10mM phosphate buffered ultrapure water at pH 6 – 11.

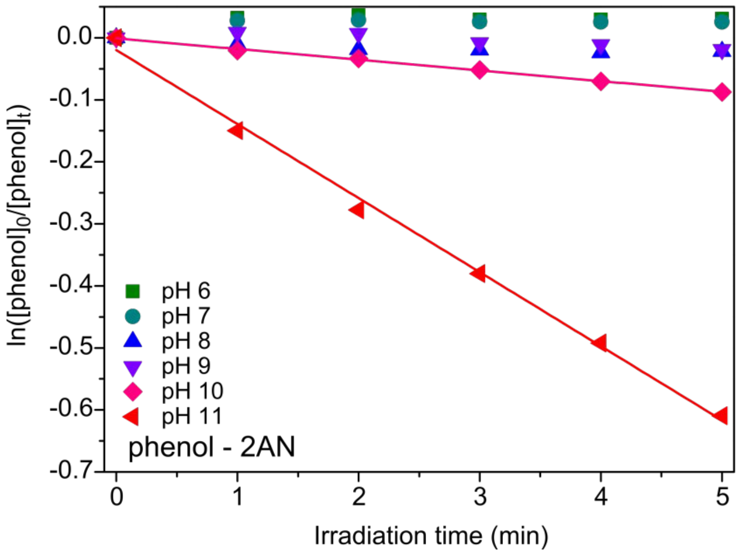

Figure S7. Logarithmic depletion kinetics of phenol (10μM) in presence of 2-acetonaphthone (25 μM) irradiated in 10mM phosphate buffered ultrapure water at pH 6 – 11.

Table S5a. Measured pseudo-first phototransformation rate constants (s^{-1}) for target compounds (all at 5μM) and phenol (10 μM) sensitized by 2-acetonaphthone (Irradiation time 300s)

Substance	[2AN] (μM)	Rate constant (s^{-1}) ± 95% confidence interval					
		pH6	pH7	pH8	pH9	pH10	pH11
aniline (Ani)	50	$(1.0±0.2)×10^{-3}$	$(1.8±0.3)×10^{-3}$	$(3.8±0.4)×10^{-3}$	$(7.1±0.6)×10^{-3}$		
4-methoxyaniline (MtA)	10	$(1.7±0.5)×10^{-3}$	$(3.4±1.0)×10^{-3}$	$(6.1±1.5)×10^{-3}$	$(7.1±1.8)×10^{-3}$	$(7.5±1.5)×10^{-3}$	$(9.4±1.5)×10^{-3}$
4-methylaniline (4MA)	10	$(1.8±0.1)×10^{-3}$	$(2.5±0.2)×10^{-3}$	$(2.7±0.1)×10^{-3}$	$(2.6±0.1)×10^{-3}$	$(3.3±0.1)×10^{-3}$	$(3.7±0.1)×10^{-3}$
N,N-dimethylaniline (DMA)	25	$(7.4±0.6)×10^{-4}$	$(1.4±0.1)×10^{-3}$	$(1.9±0.2)×10^{-3}$	$(2.0±0.2)×10^{-3}$	$(2.7±0.3)×10^{-3}$	$(7.1±0.4)×10^{-3}$
	50	$(6.3±0.4)×10^{-4}$	$(1.2±0.1)×10^{-3}$	$(1.4±0.2)×10^{-3}$	$(1.6±0.2)×10^{-3}$	$(2.1±0.2)×10^{-3}$	$(4.7±0.3)×10^{-3}$
sulfamethoxazole (SMX)	100	$(2.2±0.3)×10^{-4}$	$(2.3±0.2)×10^{-4}$	$(7.3±0.5)×10^{-4}$	$(8.3±0.5)×10^{-4}$	$(1.3±0.4)×10^{-3}$	$(1.7±0.1)×10^{-3}$
sulfachloropyridazine (SCPD)	50	$(1.4±0.2)×10^{-3}$	$(1.1±0.2)×10^{-3}$	$(9.2±0.5)×10^{-4}$	$(6.1±0.5)×10^{-4}$		
sulfadiazine (SD)	100	$(1.1±0.1)×10^{-3}$	$(1.8±0.2)×10^{-3}$	$(1.8±0.2)×10^{-3}$	$(1.2±0.1)×10^{-3}$	$(1.3±0.2)×10^{-3}$	$(1.7±0.1)×10^{-3}$
4-cyanophenol (4CNP)	25	$(1.4±0.3)×10^{-4}$	$(5.0±1.6)×10^{-4}$	$(1.6±0.3)×10^{-3}$	$(1.5±0.4)×10^{-3}$		
phenol	25	n.d.	n.d.	$(7.2±2.0)×10^{-5}$	$(8.0±3.0)×10^{-5}$	$(2.9±0.1)×10^{-4}$	$(2.0±0.1)×10^{-3}$

Table S5b. Corrected pseudo-first phototransformation rate constants (s^{-1}) for target compounds (all at 5µM) and phenol (10 µM) sensitized by 2-acetonaphthone (Irradiation time 300s)

Substance	[2AN] (µM)	Rate constant (s^{-1}) ± 95% confidence interval					
		pH6	pH7	pH8	pH9	pH10	pH11
aniline (Ani)	50	(1.1±0.2)×10^{-3}	(1.9±0.3)×10^{-3}	(3.9±0.4)×10^{-3}	(7.3±0.6)×10^{-3}		
4-methoxyaniline (MtA)	10	(1.7±0.5)×10^{-3}	(3.4±1.0)×10^{-3}	(6.2±1.5)×10^{-3}	(7.1±1.8)×10^{-3}	(7.6±1.5)×10^{-3}	(9.5±1.5)×10^{-3}
	25	(2.2±0.5)×10^{-3}	(3.4±0.8)×10^{-3}	(6.0±0.2)×10^{-3}	(9.6±0.2)×10^{-3}	(1.0±0.1)×10^{-2}	(1.3±0.1)×10^{-2}
4-methylaniline (4MA)	10	(1.8±0.1)×10^{-3}	(2.5±0.2)×10^{-3}	(2.7±0.1)×10^{-3}	(2.6±0.1)×10^{-3}	(3.4±0.1)×10^{-3}	(3.7±0.1)×10^{-3}
	25	(2.6±0.1)×10^{-3}	(4.0±0.2)×10^{-3}	(4.4±0.2)×10^{-3}	(7.5±0.1)×10^{-3}	(8.0±0.1)×10^{-3}	(7.7±0.1)×10^{-3}
N,N-dimethylaniline (DMA)	25	(7.5±0.6)×10^{-4}	(1.4±0.1)×10^{-3}	(1.9±0.2)×10^{-3}	(2.0±0.2)×10^{-3}	(2.8±0.3)×10^{-3}	(7.2±0.4)×10^{-3}
	50	(6.5±0.4)×10^{-4}	(1.2±0.1)×10^{-3}	(1.5±0.2)×10^{-3}	(1.6±0.2)×10^{-3}	(2.1±0.2)×10^{-3}	(4.8±0.3)×10^{-3}
sulfamethoxazole (SMX)	100	(2.3±0.3)×10^{-4}	(2.4±0.2)×10^{-4}	(7.7±0.5)×10^{-4}	(8.7±0.5)×10^{-4}	(1.3±0.4)×10^{-3}	(1.8±0.1)×10^{-3}
sulfachloropyridazine (SCPD)	50	(1.4±0.2)×10^{-3}	(1.1±0.2)×10^{-3}	(9.5±0.5)×10^{-4}	(6.2±0.5)×10^{-4}		
sulfadiazine (SD)	100	(1.2±0.1)×10^{-3}	(1.9±0.2)×10^{-3}	(1.9±0.2)×10^{-3}	(1.3±0.1)×10^{-3}	(1.4±0.2)×10^{-3}	(1.8±0.1)×10^{-3}
4-cyanophenol (4CNP)	25	(1.5±0.3)×10^{-4}	(5.1±1.6)×10^{-4}	(1.6±0.3)×10^{-3}	(1.5±0.4)×10^{-3}		
phenol	25	n.d.	n.d.	(7.3±2.0)×10^{-5}	(8.1±3.0)×10^{-5}	(3.0±0.1)×10^{-4}	(2.1±0.1)×10^{-3}

Table S5c. Normalized corrected phototransformation rates (s^{-1}) for target compounds (all at 5µM) and phenol (10 µM) sensitized by 2-acetonaphthone (Irradiation time 300s)

Substance	Rate constant (s^{-1}) ± 95% confidence interval					
	pH6	pH7	pH8	pH9	pH10	pH11
aniline (Ani)	1.00±0.20	1.77±0.32	3.68±0.37	6.9±0.64		
4-methoxyaniline (MtA)	1.00±0.27	1.96±0.61	3.54±0.89	4.08±1.04	4.35±0.86	5.44±0.87
	1.00±0.23	1.58±0.37	2.77±0.77	4.46±0.70	4.84±0.49	5.92±0.52
4-methylaniline (4MA)	1.00±0.07	1.41±0.10	1.52±0.07	1.47±0.03	1.90±0.02	2.08±0.03
	1.00±0.05	1.56±0.07	1.69±0.05	2.90±0.02	3.07±0.01	2.96±0.02
N,N-dimethylaniline (DMA)	1.00±0.08	1.91±0.08	2.56±0.38	2.67±0.38	3.67±0.41	9.63±0.50
	1.00±0.06	1.85±0.03	2.23±0.24	2.50±0.23	3.29±0.22	7.33±0.51
sulfamethoxazole (SMX)	1.00±0.12	1.03±0.11	3.32±0.20	3.76±0.21	5.67±0.16	7.67±0.10
sulfachloropyridazine (SCPD)	1.00±0.15	0.79±0.15	0.68±0.11	0.44±0.05		
sulfadiazine (SD)	1.00±0.06	1.66±0.15	1.63±0.19	1.10±0.06	1.23±0.10	1.52±0.04
4-cyanophenol (4CNP)	1.00±0.22	3.49±1.17	10.91±1.90	10.56±2.51		
phenol	n.d.	n.d.	n.d.	1.00±0.28	3.96±0.11	27.4±1.0

Table S6a. Measured pseudo-first phototransformation rates constants (s^{-1}) for target compounds (all at 5μM) sensitized by 4-carboxybenzophenone (Irradiation time 300s)

Substance	[CBBP] (μM)	Rate constants (s^{-1}) ± 95% confidence interval					
		pH6	pH7	pH8	pH9	pH10	pH11
aniline (Ani)							
4-methoxyaniline (MtA)	50	$(1.4±0.3)×10^{-3}$	$(1.8±0.4)×10^{-3}$	$(3.3±0.4)×10^{-3}$	$(4.4±0.3)×10^{-3}$	$(5.0±0.3)×10^{-3}$	$(6.7±0.1)×10^{-3}$
4-methylaniline (4MA)	50	$(1.7±0.1)×10^{-3}$	$(1.9±0.1)×10^{-3}$	$(2.1±0.1)×10^{-3}$	$(2.5±0.1)×10^{-3}$	$(3.8±0.1)×10^{-3}$	$(5.2±0.1)×10^{-3}$
N,N-dimethylaniline (DMA)	50	$(3.7±0.6)×10^{-4}$	$(7.2±0.5)×10^{-4}$	$(8.2±0.2)×10^{-4}$	$(8.5±0.9)×10^{-4}$	$(1.1±0.1)×10^{-3}$	$(2.3±0.1)×10^{-3}$
sulfamethoxazole (SMX)	50	$(5.2±0.2)×10^{-4}$	$(7.6±0.8)×10^{-4}$	$(1.7±0.1)×10^{-3}$	$(2.5±0.1)×10^{-4}$	$(2.7±0.1)×10^{-3}$	$(3.2±0.1)×10^{-3}$
sulfachloropyridazine (SCPD)							
sulfadiazine (SD)	50	$(2.7±0.1)×10^{-3}$	$(4.1±0.1)×10^{-3}$	$(3.9±0.3)×10^{-3}$	$(2.6±0.2)×10^{-3}$	$(2.8±0.2)×10^{-3}$	$(2.8±0.1)×10^{-3}$
4-cyanophenol (4CNP)	50	$(6.4±0.5)×10^{-4}$	$(5.5±0.5)×10^{-4}$	$(6.0±0.4)×10^{-4}$	$(5.7±0.3)×10^{-4}$	$(4.0±0.2)×10^{-4}$	$(3.2±0.2)×10^{-4}$

Table S6b. Corrected pseudo-first phototransformation rate constants (s^{-1}) for target compounds (all at 5μM) sensitized by 4-carboxybenzophenone (Irradiation time 300s)

Substance	[CBBP] (μM)	Rate constant (s^{-1}) ± 95% confidence interval					
		pH6	pH7	pH8	pH9	pH10	pH11
aniline (Ani)							
4-methoxyaniline (MtA)	50	$(1.4±0.3)×10^{-3}$	$(1.8±0.4)×10^{-3}$	$(3.3±0.4)×10^{-3}$	$(4.5±0.3)×10^{-3}$	$(5.0±0.3)×10^{-3}$	$(6.7±0.1)×10^{-3}$
4-methylaniline (4MA)	50	$(1.7±0.1)×10^{-3}$	$(1.9±0.1)×10^{-3}$	$(2.1±0.1)×10^{-3}$	$(2.5±0.1)×10^{-3}$	$(3.8±0.1)×10^{-3}$	$(5.3±0.1)×10^{-3}$
N,N-dimethylaniline (DMA)	50	$(3.7±0.6)×10^{-4}$	$(7.3±0.5)×10^{-4}$	$(8.3±0.2)×10^{-4}$	$(8.5±0.9)×10^{-4}$	$(1.1±0.1)×10^{-3}$	$(2.3±0.1)×10^{-3}$
sulfamethoxazole (SMX)	50	$(5.2±0.2)×10^{-4}$	$(7.7±0.8)×10^{-4}$	$(1.7±0.1)×10^{-3}$	$(2.5±0.1)×10^{-4}$	$(2.8±0.1)×10^{-3}$	$(3.2±0.1)×10^{-3}$
sulfachloropyridazine (SCPD)							
sulfadiazine (SD)	50	$(2.7±0.1)×10^{-3}$	$(4.2±0.1)×10^{-3}$	$(3.9±0.3)×10^{-3}$	$(2.6±0.2)×10^{-3}$	$(2.8±0.2)×10^{-3}$	$(2.8±0.1)×10^{-3}$
4-cyanophenol (4CNP)	50	$(6.4±0.5)×10^{-4}$	$(5.6±0.5)×10^{-4}$	$(6.1±0.4)×10^{-4}$	$(5.8±0.3)×10^{-4}$	$(4.1±0.2)×10^{-4}$	$(3.2±0.2)×10^{-4}$

Table S6c. Normalized corrected phototransformation rate constants (s^{-1}) for target compounds (all at 5µM) and sensitized by 4-carboxybenzophenone (Irradiation time 300s)

Substance	Normalized rate constant ± 95% confidence interval					
	pH6	pH7	pH8	pH9	pH10	pH11
aniline (Ani)						
4-methoxyaniline (MtA)	1.00±0.19	1.29±0.27	2.36±0.26	3.18±0.23	3.61±0.22	4.78±0.06
4-methylaniline (4MA)	1.00±0.06	1.13±0.06	1.26±0.03	1.49±0.03	2.31±0.02	3.17±0.08
N,N-dimethylaniline (DMA)	1.00±0.06	1.95±0.15	2.22±0.06	2.28±0.24	2.82±0.15	6.23±0.32
sulfamethoxazole (SMX)	1.00±0.04	1.48±0.15	3.31±0.19	4.75±0.14	5.27±0.05	6.17±0.09
sulfachloropyridazine (SCPD)						
sulfadiazine (SD)	1.00±0.01	1.56±0.02	1.46±0.13	0.98±0.06	1.03±0.07	1.03±0.04
4-cyanophenol (4CNP)	1.00±0.08	0.87±0.07	1.04±0.06	0.91±0.05	0.64±0.03	0.50±0.04

Table S7a. Measured pseudo-first phototransformation rate constants (s^{-1}) for target compounds (all at 5µM) sensitized by SRFA 2.5 mg C L^{-1}

Substance	Irradiation time (min)	Rate constant (s^{-1}) ± 95% confidence interval					
		pH6	pH7	pH8	pH9	pH10	pH11
aniline (Ani)	90	$(5.6±0.5)×10^{-5}$	$(6.0±0.8)×10^{-5}$	$(6.4±0.6)×10^{-5}$	$(8.7±0.8)×10^{-5}$		
4-methoxyaniline (MtA)	60	$(1.4±0.2)×10^{-4}$	$(1.9±0.2)×10^{-4}$	$(2.7±0.1)×10^{-4}$	$(2.7±0.1)×10^{-4}$	$(2.7±0.1)×10^{-4}$	$(3.2±0.1)×10^{-4}$
4-methylaniline (4MA)	60	$(5.6±0.6)×10^{-5}$	$(8.2±0.7)×10^{-5}$	$(1.3±0.1)×10^{-4}$	$(1.4±0.1)×10^{-4}$	$(1.7±0.1)×10^{-4}$	$(2.5±0.1)×10^{-4}$
N,N-dimethylaniline (DMA)	60	$(3.5±1.0)×10^{-5}$	$(4.9±0.5)×10^{-5}$	$(4.2±0.3)×10^{-5}$	$(4.9±0.2)×10^{-5}$	$(5.8±0.5)×10^{-5}$	$(7.2±0.5)×10^{-5}$
sulfamethoxazole (SMX)	60	$(1.1±0.1)×10^{-5}$	$(8.1±0.5)×10^{-6}$	$(1.1±0.1)×10^{-5}$	$(9.7±0.1)×10^{-6}$	$(1.8±0.1)×10^{-5}$	$(3.2±0.2)×10^{-5}$
sulfachloropyridazine (SCPD)							
sulfadiazine (SD)	60	$(1.2±0.1)×10^{-4}$	$(1.2±0.1)×10^{-4}$	$(5.3±0.3)×10^{-5}$	$(3.3±0.1)×10^{-5}$	$(2.8±0.1)×10^{-5}$	$(3.8±0.1)×10^{-5}$
4-cyanophenol (4CNP)							

Table S7b. Corrected pseudo-first phototransformation rate constants (s^{-1}) for target compounds (all at 5μM) sensitized by SRFA 2.5 mg C L^{-1}

Substance	Irradiation time (min)	Rate constant (s^{-1}) ± 95% confidence interval					
		pH6	pH7	pH8	pH9	pH10	pH11
aniline (Ani)	90	$(5.8±0.5)×10^{-5}$	$(6.3±0.8)×10^{-5}$	$(6.6±0.6)×10^{-5}$	$(9.1±0.8)×10^{-5}$		
4-methoxyaniline (MtA)	60	$(1.5±0.2)×10^{-4}$	$(2.0±0.2)×10^{-4}$	$(2.8±0.1)×10^{-4}$	$(2.8±0.1)×10^{-4}$	$(2.8±0.1)×10^{-4}$	$(3.4±0.1)×10^{-4}$
4-methylaniline (4MA)	60	$(5.8±0.6)×10^{-5}$	$(8.6±0.7)×10^{-5}$	$(1.3±0.1)×10^{-4}$	$(1.5±0.1)×10^{-4}$	$(1.7±0.1)×10^{-4}$	$(2.7±0.1)×10^{-4}$
N,N-dimethylaniline (DMA)	60	$(3.6±1.0)×10^{-5}$	$(5.1±0.5)×10^{-5}$	$(4.4±0.3)×10^{-5}$	$(5.1±0.2)×10^{-5}$	$(6.1±0.5)×10^{-5}$	$(7.5±0.5)×10^{-5}$
sulfamethoxazole (SMX)	60	$(1.2±0.1)×10^{-5}$	$(8.4±0.5)×10^{-6}$	$(1.1±0.1)×10^{-5}$	$(1.0±0.1)×10^{-5}$	$(1.9±0.1)×10^{-5}$	$(3.4±0.2)×10^{-5}$
sulfachloropyridazine (SCPD)							
sulfadiazine (SD)	60	$(1.2±0.1)×10^{-4}$	$(1.2±0.1)×10^{-4}$	$(5.6±0.3)×10^{-5}$	$(3.4±0.1)×10^{-5}$	$(3.0±0.1)×10^{-5}$	$(4.0±0.1)×10^{-5}$
4-cyanophenol (4CNP)							

Table S7c. Normalized corrected phototransformation rate constants (s^{-1}) for target compounds (all at 5μM) sensitized by SRFA 2.5 mg C L^{-1}

Substance	Normalized rate constants ± 95% confidence interval					
	pH6	pH7	pH8	pH9	pH10	pH11
aniline (Ani)	1.00±0.08	1.09±0.14	1.15±0.10	1.56±0.14		
4-methoxyaniline (MtA)	1.00±0.13	1.36±0.16	1.92±0.09	1.89±0.02	1.91±0.03	2.27±0.06
4-methylaniline (4MA)	1.00±0.11	1.49±0.12	2.27±0.06	2.52±0.07	3.02±0.05	4.58±0.13
N,N-dimethylaniline (DMA)	1.00±0.30	1.42±0.15	1.23±0.09	1.43±0.05	1.68±0.14	2.10±0.15
sulfamethoxazole (SMX)	1.00±0.09	0.70±0.04	0.95±0.03	0.84±0.09	1.61±0.09	2.81±0.15
sulfachloropyridazine (SCPD)						
sulfadiazine (SD)	1.00±0.07	0.97±0.04	0.44±0.03	0.27±0.01	0.23±0.01	0.31±0.01
4-cyanophenol (4CNP)						

Table S8a. Measured pseudo-first phototransformation rate constant (s^{-1}) for target compounds (all at 5μM) sensitized by PLFA 2.5 mg C L^{-1}

Substance	Irradiation time (min)	Rate constant (s^{-1}) ± 95% confidence interval					
		pH6	pH7	pH8	pH9	pH10	pH11
aniline (Ani)	90	$(6.2±1.2)×10^{-5}$	$(7.8±1.2)×10^{-5}$	$(1.4±0.1)×10^{-4}$	$(3.0±0.2)×10^{-4}$		
4-methoxyaniline (MtA)	60	$(2.2±0.2)×10^{-4}$	$(3.4±0.4)×10^{-4}$	$(5.3±0.3)×10^{-4}$	$(5.2±0.2)×10^{-4}$	$(5.7±0.1)×10^{-4}$	$(8.6±0.5)×10^{-4}$
4-methylaniline (4MA)	60	$(1.1±0.1)×10^{-5}$	$(1.8±0.2)×10^{-4}$	$(2.7±0.1)×10^{-4}$	$(3.7±0.1)×10^{-4}$	$(6.7±0.3)×10^{-4}$	$(7.2±0.1)×10^{-4}$
N,N-dimethylaniline (DMA)	60	$(1.6±0.1)×10^{-4}$	$(2.2±0.1)×10^{-4}$	$(3.1±0.3)×10^{-5}$			
sulfamethoxazole (SMX)	60	$(2.1±0.1)×10^{-5}$	$(1.2±0.2)×10^{-5}$	$(3.6±0.3)×10^{-5}$	$(4.8±0.4)×10^{-5}$	$(6.3±0.9)×10^{-5}$	$(7.6±0.7)×10^{-5}$
sulfachloropyridazine (SCPD)							
sulfadiazine (SD)	60	$(2.6±0.3)×10^{-4}$	$(3.0±0.2)×10^{-4}$	$(2.2±0.1)×10^{-4}$	$(1.1±0.1)×10^{-4}$	$(6.7±0.3)×10^{-5}$	$(7.9±1.3)×10^{-5}$
4-cyanophenol (4CNP)							

Table S8b. Corrected pseudo-first phototransformation rate constants (s^{-1}) for target compounds (all at 5μM) sensitized by PLFA 2.5 mg C L^{-1}

Substance	Irradiation time (min)	Rate constant (s^{-1}) ± 95% confidence interval					
		pH6	pH7	pH8	pH9	pH10	pH11
aniline (Ani)	90	$(6.3±1.2)×10^{-5}$	$(8.0±1.2)×10^{-5}$	$(1.4±0.1)×10^{-4}$	$(3.1±0.2)×10^{-4}$		
4-methoxyaniline (MtA)	60	$(2.3±0.2)×10^{-4}$	$(3.5±0.4)×10^{-4}$	$(5.5±0.3)×10^{-4}$	$(5.3±0.2)×10^{-4}$	$(5.9±0.1)×10^{-4}$	$(8.8±0.5)×10^{-4}$
4-methylaniline (4MA)	60	$(1.1±0.1)×10^{-5}$	$(1.9±0.2)×10^{-4}$	$(2.8±0.1)×10^{-4}$	$(3.8±0.1)×10^{-4}$	$(6.9±0.3)×10^{-4}$	$(7.4±0.1)×10^{-4}$
N,N-dimethylaniline (DMA)	60	$(1.7±0.1)×10^{-4}$	$(2.2±0.1)×10^{-4}$	$(3.1±0.3)×10^{-5}$			
sulfamethoxazole (SMX)	60	$(2.1±0.1)×10^{-5}$	$(1.2±0.2)×10^{-5}$	$(3.7±0.3)×10^{-5}$	$(4.9±0.4)×10^{-5}$	$(6.5±0.9)×10^{-5}$	$(7.9±0.7)×10^{-5}$
sulfachloropyridazine (SCPD)							
sulfadiazine (SD)	60	$(2.7±0.3)×10^{-4}$	$(3.1±0.2)×10^{-4}$	$(2.2±0.1)×10^{-4}$	$(1.2±0.1)×10^{-4}$	$(6.8±0.3)×10^{-5}$	$(8.1±1.3)×10^{-5}$
4-cyanophenol (4CNP)							

Table S8c. Normalized corrected phototransformation rate constants (s^{-1}) for target compounds (all at 5μM) sensitized by PLFA 2.5 mg C L^{-1}

Substance	Normalized rate constant ± 95% confidence interval					
	pH6	pH7	pH8	pH9	pH10	pH11
aniline (Ani)	1.00±0.20	1.26±0.19	2.26±0.19	4.85±0.30		
4-methoxyaniline (MtA)	1.00±0.11	1.55±0.18	2.43±0.14	2.35±0.09	2.60±0.03	3.91±0.21
4-methylaniline (4MA)	1.00±0.11	1.73±0.14	2.54±0.07	3.51±0.07	6.35±0.26	6.79±0.12
N,N-dimethylaniline (DMA)	1.00±0.01	1.32±0.03				
sulfamethoxazole (SMX)	1.00±0.06	0.56±0.09	1.77±0.15	2.34±0.21	3.07±0.46	3.71±0.35
sulfachloropyridazine (SCPD)						
sulfadiazine (SD)	1.00±0.13	1.15±0.08	0.82±0.03	0.42±0.02	0.25±0.01	0.30±0.05
4-cyanophenol (4CNP)						

Table S9. Light attenuation factors for SRFA/PLFA at 2.5 mg C L^{-1} for pH 6 – 11

pH	SRFA	PLFA
6	0.962	0.977
7	0.960	0.976
8	0.960	0.975
9	0.956	0.975
10	0.954	0.973
11	0.950	0.972

Table S10. pH-independent light attenuation factors for 2AN/CBBP at different concentrations

Concentration (μM)	2AN	CBBP
10	0.995	0.999
25	0.987	0.997
50	0.974	0.995
100	0.949	0.990

Table S11. Light attenuation factors for fulvic acid (2.5 mg C L^{-1}) model photosensitizer mixtures (10μM)

pH	2AN/SRFA	2AN/PLFA	CBBP/SRFA	CBBP/PLFA
6	0.951	0.965	0.960	0.975
7	0.949	0.964	0.958	0.974
8	0.949	0.964	0.958	0.973
9	0.946	0.963	0.955	0.973
10	0.943	0.962	0.952	0.972
11	0.939	0.960	0.948	0.970

Table S12. Light attenuation factors for fulvic acid (2.5 mg C L^{-1}) model photosensitizer mixtures (25µM)

pH	2AN/SRFA	2AN/PLFA	CBBP/SRFA	CBBP/PLFA
6	0.935	0.949	0.958	0.972
7	0.933	0.948	0.955	0.971
8	0.933	0.947	0.955	0.971
9	0.930	0.947	0.952	0.970
10	0.927	0.946	0.950	0.969
11	0.923	0.944	0.945	0.967

Table S13. Light attenuation factors for fulvic acid (2.5 mg C L^{-1}) model photosensitizer mixtures (50µM)

pH	2AN/SRFA	2AN/PLFA	CBBP/SRFA	CBBP/PLFA
6	0.910	0.923	0.953	0.968
7	0.908	0.922	0.951	0.967
8	0.908	0.922	0.951	0.966
9	0.905	0.922	0.948	0.966
10	0.903	0.920	0.946	0.964
11	0.899	0.919	0.941	0.963

Table S14. Light attenuation factors for fulvic acid (2.5 mg C L^{-1}) model photosensitizer mixtures (100µM)

pH	2AN/SRFA	2AN/PLFA	CBBP/SRFA	CBBP/PLFA
6	0.865	0.877	0.945	0.959
7	0.863	0.876	0.943	0.958
8	0.863	0.876	0.942	0.958
9	0.860	0.875	0.939	0.957
10	0.858	0.874	0.937	0.956
11	0.854	0.873	0.933	0.954

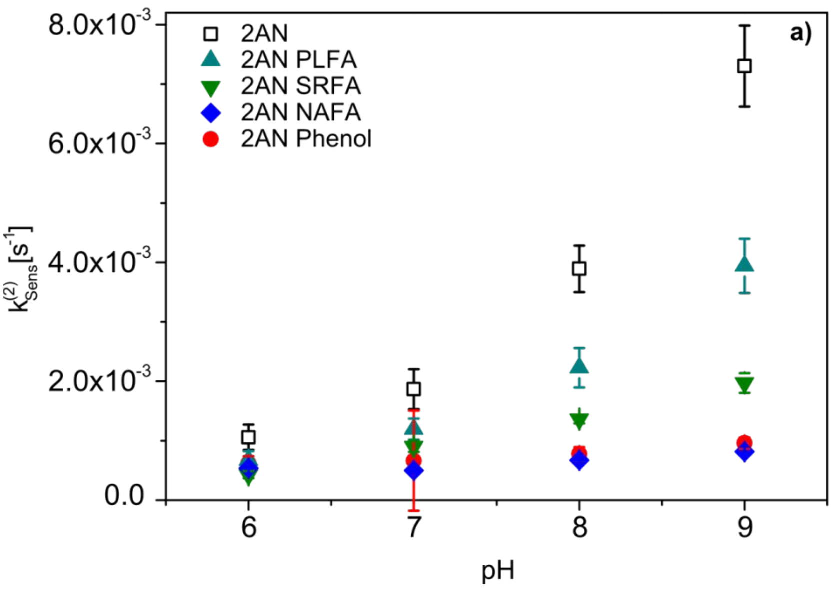

Figure S8. Corrected pseudo-first order phototransformation rates of aniline (5 µM) sensitized by 2AN (50 µM) in presence of different fulvic acids (2.5 mg C L^{-1}) and the model antioxidant phenol (10 µM), respectively.

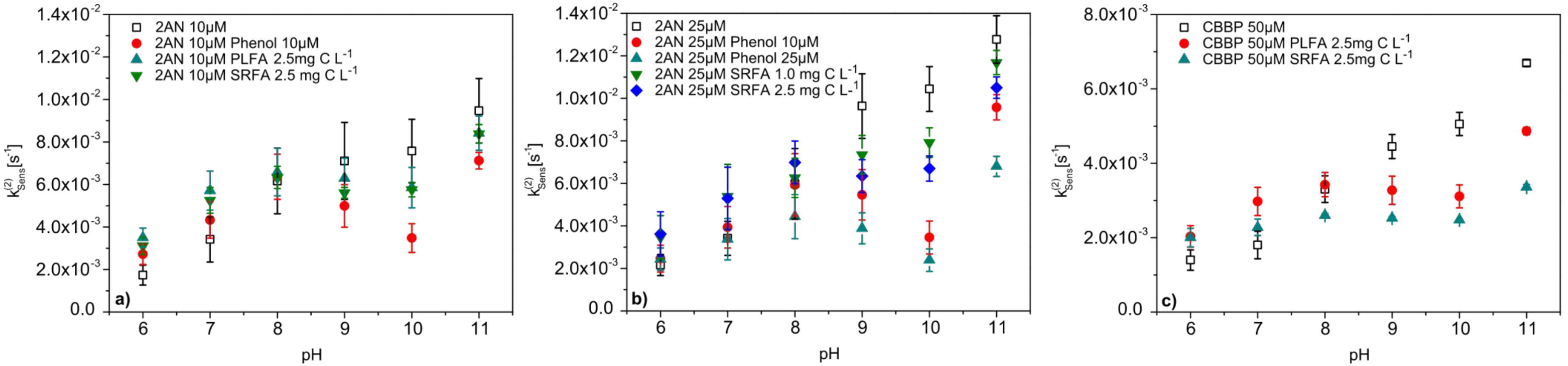

Figure S9. (a) Corrected pseudo-first order phototransformation rates of 4-methoxyaniline (5 µM) sensitized by 10 µM 2AN (a), 25 µM 2AN (b) and (c) CBBP (50 µM) in presence fulvic acids and the model antioxidant phenol, respectively.

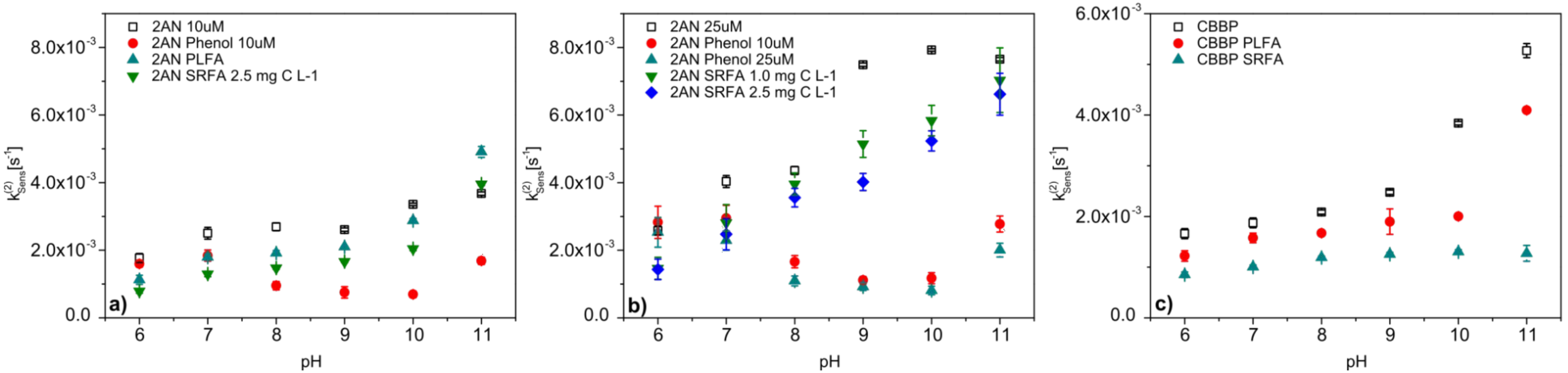

Figure S10. (a) Corrected pseudo-first order phototransformation rates of 4-methylaniline (5 µM) sensitized by 10 µM 2AN (a), 25 µM 2AN (b) and (c) CBBP (50 µM) in presence fulvic acids and the model antioxidant phenol, respectively.

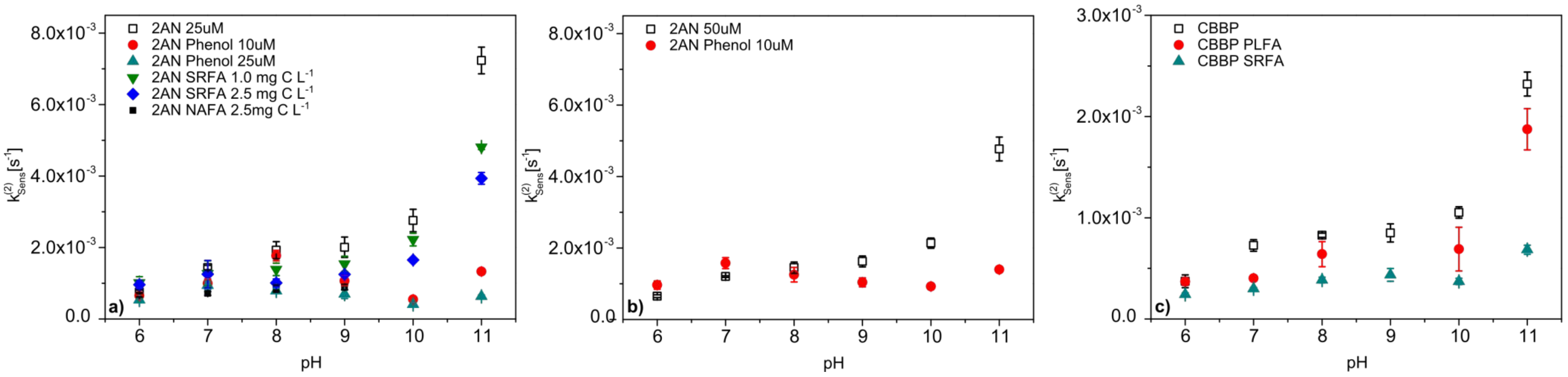

Figure S11. (a) Corrected pseudo-first order phototransformation rates of *N,N*-DMA (5 µM) sensitized by 25 µM 2AN (a), 50 µM 2AN (b) and (c) CBBP (50 µM) in presence fulvic acids and the model antioxidant phenol, respectively.

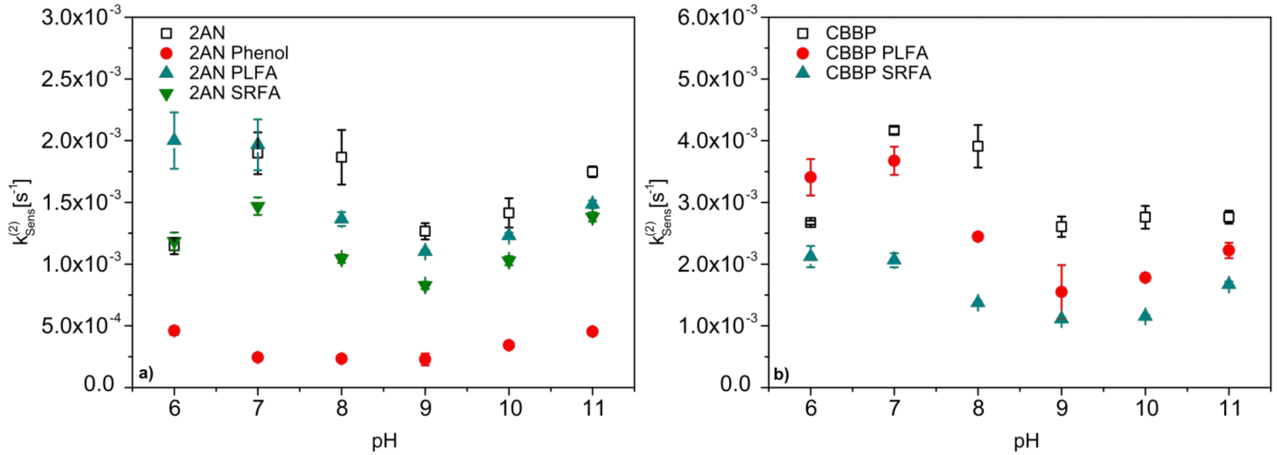

Figure S12. (a) Corrected pseudo-first order phototransformation rates of SD (5 µM) sensitized by 100 µM 2AN (a) and (b) 50 µM CBBP in presence fulvic acids and the model antioxidant phenol, respectively.

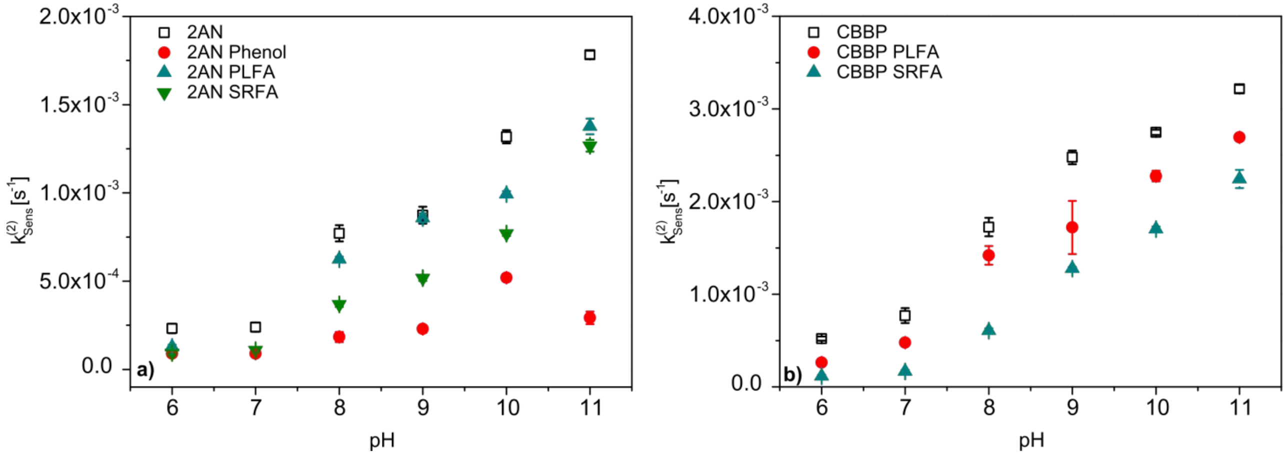

Figure S13. (a) Corrected pseudo-first order phototransformation rates of SMX (5 μM) sensitized by 100 μM 2AN (a) and (b) 50 μM CBBP in presence fulvic acids and the model antioxidant phenol, respectively.

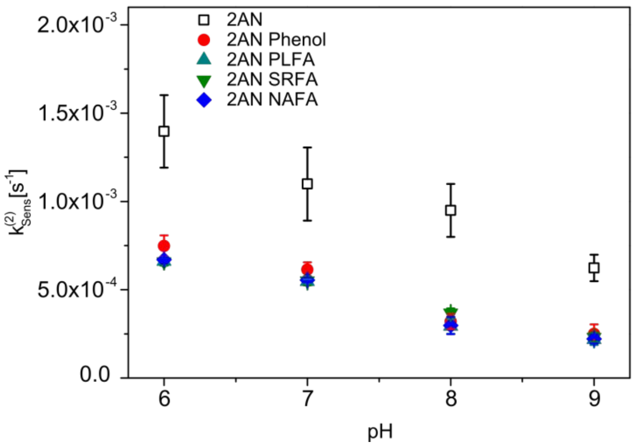

Figure S14. (a) Corrected pseudo-first order phototransformation rates of SCPD (5 μM) sensitized by 50 μM 2AN in presence fulvic acids and the model antioxidant phenol, respectively.

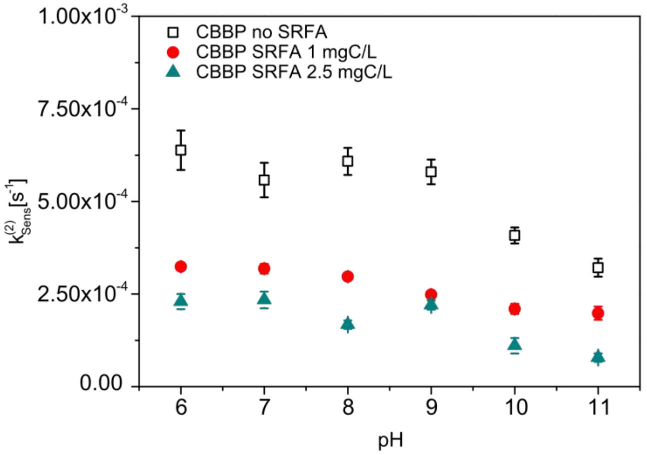

Figure S15. (a) Corrected pseudo-first order phototransformation rates of 4CNP (5 µM) sensitized by 50 µM CBBP in presence fulvic acids and the model antioxidant phenol, respectively.

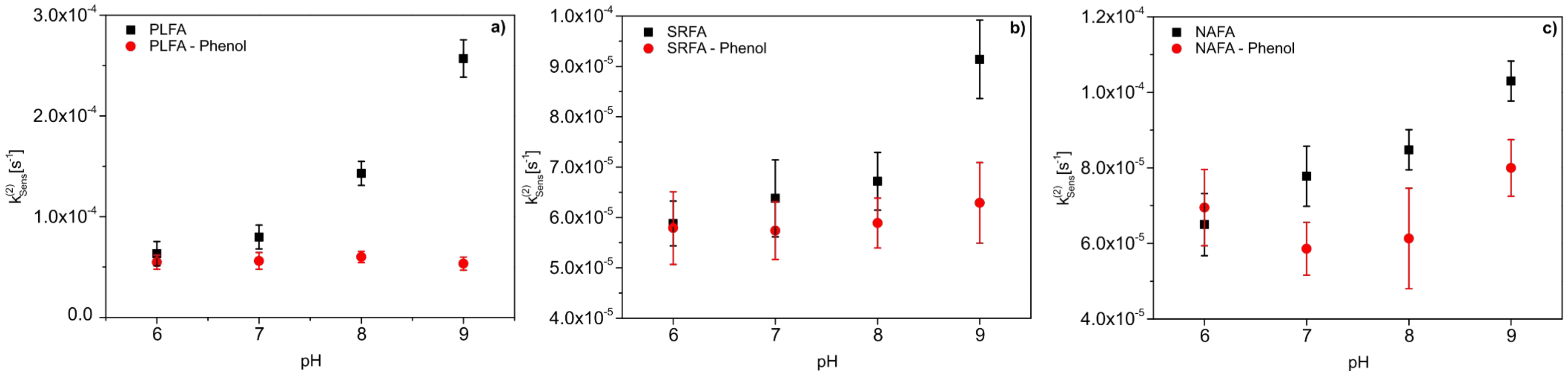

Figure S16. Corrected pseudo-first order phototransformation rates of aniline (5 µM) sensitized by different fulvic acids (a) PLFA, (b) SRFA, (c) NAFA, all 5 mg C L^{-1} w/wo the model antioxidant phenol (10 µM) and (d) the calculated inhibition efficiency ($1-k_{Sens,AO}/k_{Sens}$).

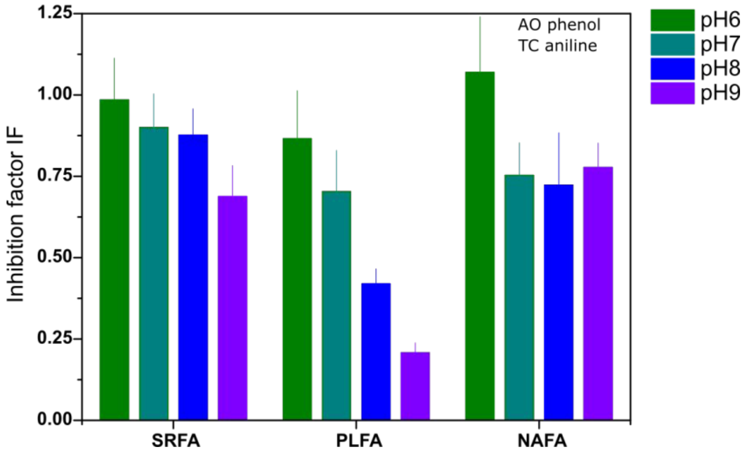

Figure S17. Inhibition factors for the DOM-photosensitized transformation of aniline in the presence of 10 µM phenol as a model antioxidant, [DOM] = 2.5 mg C L^{-1}.

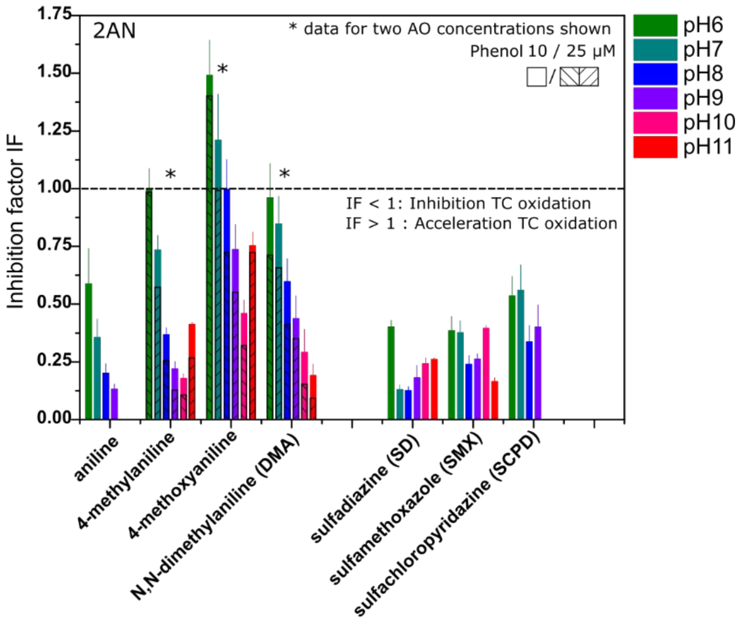

Figure S18. Inhibition factors of photosensitized transformation of target compounds (TCs) for model photosensitizer 2AN with the model antioxidant phenol added.

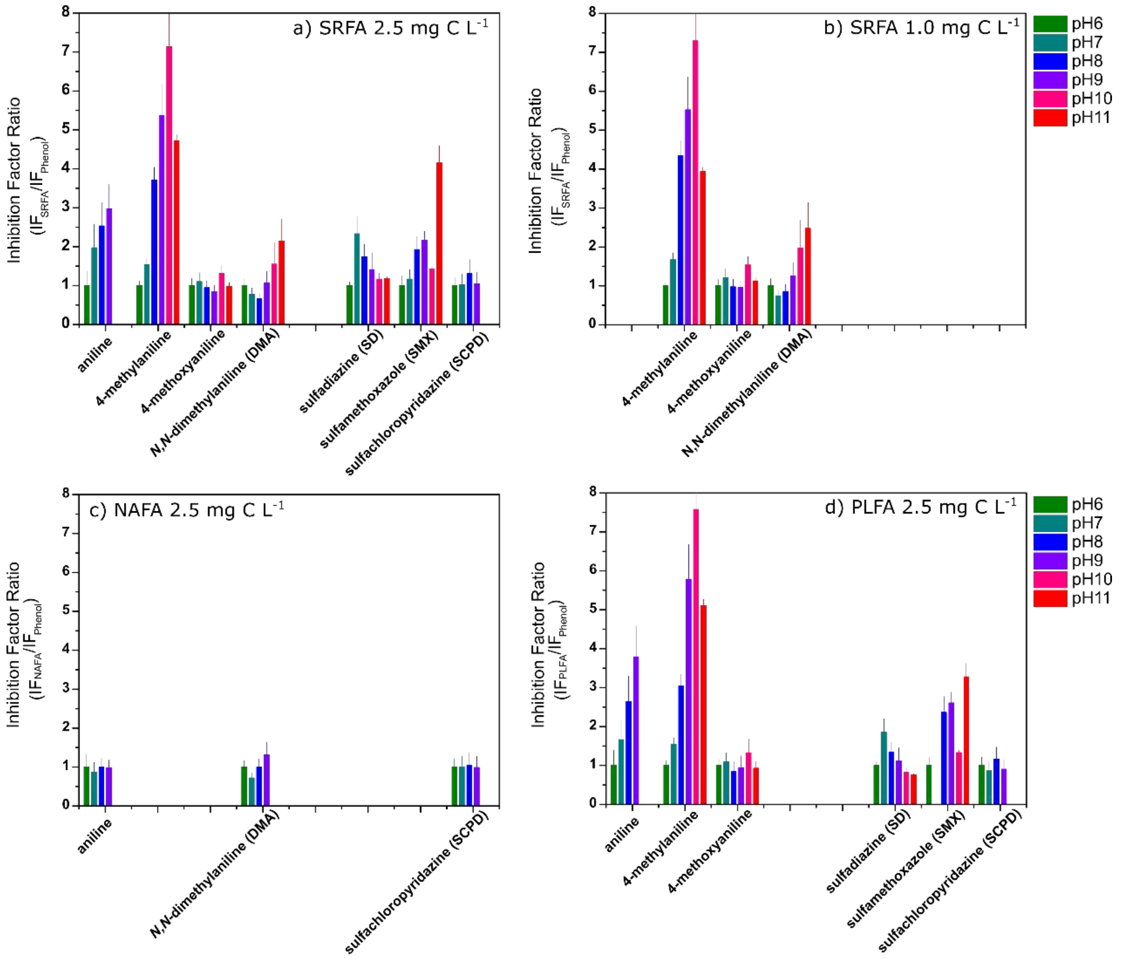

Figure S19. Inhibition factor ratios of DOMs and model antioxidant phenol for TC transformation sensitized with 2AN. (a) IF_{SRFA} [2.5 mg C L^{-1}] / IF_{Phenol} [10μM]; (b) IF_{SRFA} [1.0 mg C L^{-1}] / IF_{Phenol} [10μM]; (c) IF_{NAFA} [2.5 mg C L^{-1}] / IF_{Phenol} [10μM]; (d) IF_{PLFA} [2.5 mg C L^{-1}] / IF_{Phenol} [10μM].

Text S6. Dependence of redox potentials of phenol and anilines on pH.

To assess the driving force of the one-electron oxidation of phenol by anilines, we consider here the half-cell reduction potentials of the corresponding radicals.
For phenol (PhOH), the following half-cell reaction is considered:

$$PhO^{\bullet} + e^{-} + H^{+} \rightleftharpoons PhOH \qquad \textbf{(Sa1)}$$

where $PhO^{\bullet}$ is phenoxyl radical. The standard reduction potential for the half-cell reaction involving phenoxide anion (PhO^{-}):

$$PhO^{\bullet} + e^{-} \rightleftharpoons PhO^{-} \qquad \textbf{(Sa2)}$$

is defined here as $E^{0}_{\mathrm{red}}(PhO^{\bullet}/PhO^{-})$, and is known from the literature to be 0.79 V vs standard hydrogen electrode (SHE).[29] Applying Nernst equation for a temperature of 25 °C (298 K) to the reaction described by Eq. Sa1, one obtains:

$$E_{\mathrm{red}}(PhO^{\bullet},\mathrm{pH}) / \mathrm{V} = E^{0}_{\mathrm{red}}(PhO^{\bullet}/PhO^{-}) / \mathrm{V} + 0.059 \times \log\left(1 + 10^{\mathrm{p}K_{\mathrm{a}} - \mathrm{pH}}\right) \qquad \textbf{(Sa3)}$$

where log is the decadic logarithm and $\mathrm{p}K_{\mathrm{a}}$ is the negative decadic logarithm for the proton dissociation constant of PhOH (see Table 1 of the main paper). $E_{\mathrm{red}}(PhO^{\bullet},\mathrm{pH})$ is plotted vs pH in Figure 4a of the main paper. The function consists of a basically constant term (equal to $E^{0}_{\mathrm{red}}(PhO^{\bullet}/PhO^{-})$) for pH >> $\mathrm{p}K_{\mathrm{a}}$, and an oblique line with slope −0.059 V for pH << $\mathrm{p}K_{\mathrm{a}}$, and a non-linear portion for pH values close to $\mathrm{p}K_{\mathrm{a}}$.

For the anilines (R-$PhNH_2$), the treatment of the pH-dependent reduction potential is analogous as for phenols, but one has also to consider the deprotonation reaction of the aniline radical cation (R-$PhNH_2^{\bullet+}$) (see the $\mathrm{p}K_{\mathrm{a}}$ values, defined as $\mathrm{p}K_{\mathrm{a}}^{*}$, in Table 1 of the main paper).
For the half-cell reaction:

$$\text{R-}PhNH_2^{\bullet+} + e^{-} \rightleftharpoons \text{R-}PhNH_2 \qquad \textbf{(Sa4)}$$

the standard one electron reduction potential (E^0_{red}(R-PhNH$_2^{\bullet+}$/R-PhNH$_2$)) is known from the literature (see Table 1 of the main paper). The following half-cell reactions have to be considered to describe the pH dependence of the reduction potential:

$$\text{R-PhNH}_2^{\bullet+} + e^- + H^+ \rightleftharpoons \text{R-PhNH}_3^+ \quad \textbf{(Sa5)}$$

$$\text{R-PhNH}^{\bullet} + e^- + H^+ \rightleftharpoons \text{R-PhNH}_2 \quad \textbf{(Sa6)}$$

Applying Nernst equation to reactions Sa5 and Sa6 yields the reduction potential of the aniline as a function of pH:

$$E_{\mathrm{red}}(\text{R-PhNH}_2^{\bullet},\mathrm{pH}) / \mathrm{V} = E^0_{\mathrm{red}}(\text{R-PhNH}_2^{\bullet+}/\text{R-PhNH}_2) / \mathrm{V} + 0.059 \times \log\left(1 + 10^{\mathrm{p}K_\mathrm{a} - \mathrm{pH}}\right) - 0.059 \times \log\left(1 + 10^{\mathrm{pH} - pK_a{}^*}\right) \quad \textbf{(Sa7)}$$

where pK_a refers here to the deprotonation of the anilinium ion. E_{red}(R-PhNH$_2^{\bullet}$,pH) is plotted vs pH in Figure 4a of the main paper. The function consists of a nearly flat plateau (equal to E^0_{red}(R-PhNH$_2^{\bullet+}$/R-PhNH$_2$)) for pK_a <pH < pK_a^*, two oblique lines with slope −0.059 V for pH << pK_a and pH >> pK_a^*, and a non-linear portions for pH values close to pK_a and pK_a^*. For *N,N*-dimehtylaniline (DMA), which does not have any exchangeable proton attached to the nitrogen atom, the last term in Eq. Sa7 disappears.
This results in the following equation:

$$E_{\mathrm{red}}(\mathrm{DMA}^{\bullet+},\mathrm{pH}) / \mathrm{V} = E^0_{\mathrm{red}}(\mathrm{DMA}^{\bullet+}/\mathrm{DMA}) / \mathrm{V} + 0.059 \times \log\left(1 + 10^{\mathrm{p}K_\mathrm{a} - \mathrm{pH}}\right) \quad \textbf{(Sa8)}$$

which is fully analogous to the function for phenol (Eq. Sa3).

References

1. Wenk, J.; Canonica, S., Phenolic antioxidants inhibit the triplet-induced transformation of anilines and sulfonamide antibiotics in aqueous solution. *Environmental Science & Technology* **2012,** *46*, (10), 5455-5462.
2. Wenk, J.; von Gunten, U.; Canonica, S., Effect of dissolved organic matter on the transformation of contaminants induced by excited triplet states and the hydroxyl radical. *Environmental Science & Technology* **2011,** *45*, (4), 1334-1340.
3. Tang, H.; Li, J.; Bie, Y.; Zhu, L.; Zou, J., Photochemical removal of aniline in aqueous solutions: Switching from photocatalytic degradation to photo-enhanced polymerization recovery. *Journal of Hazardous Materials* **2010,** *175*, (1-3), 977-984.
4. Corrochano, P.; Nachtigallová, D.; Klán, P., Photooxidation of Aniline Derivatives Can Be Activated by Freezing Their Aqueous Solutions. *Environmental Science and Technology* **2017,** *51*, (23), 13763-13770.
5. Stejskal, J.; Sapurina, I.; Trchová, M.; Konyushenko, E. N., Oxidation of aniline: Polyaniline granules, nanotubes, and oligoaniline microspheres. *Macromolecules* **2008,** *41*, (10), 3530-3536.
6. Dryer, D. J.; Korshin, G. V.; Fabbricino, M., In situ examination of the protonation behavior of fulvic acids using differential absorbance spectroscopy. *Environmental Science and Technology* **2008,** *42*, (17), 6644-6649.
7. Pace, M. L.; Reche, I.; Cole, J. J.; Fernández-Barbero, A.; Mazuecos, I. P.; Prairie, Y. T., pH change induces shifts in the size and light absorption of dissolved organic matter. *Biogeochemistry* **2012,** *108*, (1-3), 109-118.
8. Patel-Sorrentino, N.; Mounier, S.; Benaim, J. Y., Excitation-emission fluorescence matrix to study pH influence on organic matter fluorescence in the Amazon basin rivers. *Water Research* **2002,** *36*, (10), 2571-2581.
9. Canonica, S.; Jans, U.; Stemmler, K.; Hoigné, J., Transformation kinetics of phenols in water - Photosensitation by dissolved natural organic matter and aromatic ketones. *Environmental Science & Technology* **1995,** *29*, (7), 1822-1831.
10. Leresche, F.; Ludvíková, L.; Heger, D.; Klán, P.; Von Gunten, U.; Canonica, S., Laser flash photolysis study of the photoinduced oxidation of 4-(dimethylamino)benzonitrile (DMABN). *Photochemical and Photobiological Sciences* **2019,** *18*, (2), 534-545.
11. Jonsson, M.; Lind, J.; Reitberger, T.; Eriksen, T. E.; Merényi, G., Free radical combination reactions involving phenoxyl radicals. *Journal of Physical Chemistry* **1993,** *97*, (31), 8229-8233.
12. Yuan, C.; Chakraborty, M.; Canonica, S.; Weavers, L. K.; Hadad, C. M.; Chin, Y. P., Isoproturon Reappearance after Photosensitized Degradation in the Presence of Triplet Ketones or Fulvic Acids. *Environmental science & technology* **2016,** **50**, (22), 12250-12257.
13. Zafiriou, O. C., Chemistry of superoxide ion-radical (O^{2-}) in seawater. I. pK_{asw}^{*} (HOO) and uncatalyzed dismutation kinetics studied by pulse radiolysis. *Mar. Chem.* **1990,** *30*, (C), 31-43.
14. Goldstone, J. V.; Voelker, B. M., Chemistry of superoxide radical in seawater: CDOM associated sink of superoxide in coastal waters. *Environmental Science and Technology* **2000,** *34*, (6), 1043-1048.
15. Vione, D.; Minella, M.; Maurino, V.; Minero, C., Indirect photochemistry in sunlit surface waters: Photoinduced production of reactive transient species. *Chemistry - A European Journal* **2014,** *20*, (34), 10590-10606.
16. Millero, F. J.; Huang, F., Solubility of oxygen in aqueous solutions of KCl, K_2SO_4, and $CaCl_2$ as a function of concentration and temperature. *Journal of Chemical and Engineering Data* **2003,** *48*, (4), 1050-1054.

17. Millero, F. J.; Huang, F.; Graham, T. B., Solubility of oxygen in some 1-1, 2-1, 1-2, and 2-2 electrolytes as a function of concentration at 25°C. *Journal of Solution Chemistry* **2003,** *32*, (6), 473-487.
18. Arnbjerg, J.; Johnsen, M.; Nielsen, C. B.; Jørgensen, M.; Ogilby, P. R., Effect of sensitizer protonation on singlet oxygen production in aqueous and nonaqueous media. *Journal of Physical Chemistry A* **2007,** *111*, (21), 4573-4583.
19. Dalrymple, R. M.; Carfagno, A. K.; Sharpless, C. M., Correlations between dissolved organic matter optical properties and quantum yields of singlet oxygen and hydrogen peroxide. *Environmental Science and Technology* **2010,** *44*, (15), 5824-5829.
20. Ratti, M.; Canonica, S.; McNeill, K.; Bolotin, J.; Hofstetter, T. B., Isotope Fractionation Associated with the Indirect Photolysis of Substituted Anilines in Aqueous Solution. *Environmental Science and Technology* **2015,** *49*, (21), 12766-12773.
21. Haag, W. R.; Hoigne, J. In Degradatio of compounds in water by singlet oxygen, Water Chlorination: Environmental Impact and Health Effects; **1986**; pp 1011-1020.
22. Leresche, F.; Von Gunten, U.; Canonica, S., Probing the Photosensitizing and Inhibitory Effects of Dissolved Organic Matter by Using N,N-dimethyl-4-cyanoaniline (DMABN). *Environmental Science and Technology* **2016,** *50*, (20), 10997-11007.
23. Davis, C. A.; Erickson, P. R.; McNeill, K.; Janssen, E. M. L., Environmental photochemistry of fenamate NSAIDs and their radical intermediates. *Environmental Science: Processes and Impacts* **2017,** *19*, (5), 656-665.
24. Boreen, A. L.; Arnold, W. A.; McNeill, K., Triplet-sensitized photodegradation of sulfa drugs containing six-membered heterocyclic groups: Identification of an SO_2 extrusion photoproduct. *Environmental Science and Technology* **2005,** *39*, (10), 3630-3638.
25. Boreen, A. L.; Arnold, W. A.; McNeill, K., Photochemical fate of sulfa drugs in then aquatic environment: Sulfa drugs containing five-membered heterocyclic groups. *Environmental Science and Technology* **2004,** *38*, (14), 3933-3940.
26. Tratnyek, P. G.; Hoigne, J., Oxidation of substituted phenols in the environment: a QSAR analysis of rate constants for reaction with singlet oxygen. *Environmental Science & Technology* **1991,** *25*, (9), 1596-1604.
27. Janssen, E. M. L.; Erickson, P. R.; McNeill, K., Dual roles of dissolved organic matter as sensitizer and quencher in the photooxidation of tryptophan. *Environmental Science and Technology* **2014,** *48*, (9), 4916-4924.
28. Remucal, C. K.; McNeill, K., Photosensitized amino acid degradation in the presence of riboflavin and its derivatives. *Environmental Science and Technology* **2011,** *45*, (12), 5230-5237.
29. Lind, J.; Shen, X.; Eriksen, T. E.; Merényi, G., The One-Electron Reduction Potential of 4-Substituted Phenoxyl Radicals in Water. *Journal of the American Chemical Society* **1990,** *112*, (2), 479-482.
